# Characterization of Hypothalamic MCH Neuron Development in a 3D Differentiation System of Mouse Embryonic Stem Cells

**DOI:** 10.1523/ENEURO.0442-21.2022

**Published:** 2022-04-26

**Authors:** Yu Kodani, Miho Kawata, Hidetaka Suga, Yoko S. Kaneko, Akira Nakashima, Toshiki Kameyama, Kanako Saito, Hiroshi Nagasaki

**Affiliations:** 1 Department of Physiology, Fujita Health University School of Medicine, Toyoake, Aichi 470-1192, Japan; 2 Department of Endocrinology and Diabetes, Nagoya University Graduate School of Medicine, Nagoya, Aichi 466-8550, Japan; 3 Department of Physiological Chemistry, Fujita Health University School of Medicine, Toyoake, Aichi 470-1192, Japan

**Keywords:** 3D culture, ES cells, hypothalamus, MCH, neuronal differentiation

## Abstract

Hypothalamic melanin-concentrating hormone (MCH) neurons are important regulators of multiple physiological processes, such as sleep, feeding, and memory. Despite the increasing interest in their neuronal functions, the molecular mechanism underlying MCH neuron development remains poorly understood. We report that a three-dimensional culture of mouse embryonic stem cells (mESCs) can generate hypothalamic-like tissues containing MCH-positive neurons, which reproduce morphologic maturation, neuronal connectivity, and neuropeptide/neurotransmitter phenotype of native MCH neurons. Using this *in vitro* system, we demonstrate that Hedgehog (Hh) signaling serves to produce major neurochemical subtypes of MCH neurons characterized by the presence or absence of cocaine- and amphetamine-regulated transcript (CART). Without exogenous Hh signals, mESCs initially differentiated into dorsal hypothalamic/prethalamic progenitors and finally into MCH^+^CART^+^ neurons through a specific intermediate progenitor state. Conversely, activation of the Hh pathway specified ventral hypothalamic progenitors that generate both MCH^+^CART^−^ and MCH^+^CART^+^ neurons. These results suggest that *in vivo* MCH neurons may originate from multiple cell lineages that arise through early dorsoventral patterning of the hypothalamus. Additionally, we found that Hh signaling supports the differentiation of mESCs into orexin/hypocretin neurons, a well-defined cell group intermingled with MCH neurons in the lateral hypothalamic area (LHA). The present study highlights and improves the utility of mESC culture in the analysis of the developmental programs of specific hypothalamic cell types.

## Significance Statement

A growing body of literature has revealed the importance of hypothalamic melanin-concentrating hormone (MCH) neurons in energy homeostasis and the cognitive function, but their developmental biology remains relatively unknown. To establish a new approach for addressing this issue, we tested the ability of an *in vitro* differentiation system of mouse embryonic stem cells (mESCs) to recapitulate the development of MCH neurons. The mESC culture robustly generated MCH-positive neurons resembling native neurons in several aspects and provided evidence that Hedgehog (Hh) signaling is a key factor to produce neurochemical subtypes of MCH neurons. Our results demonstrate the suitability of mESC culture as a platform to study the molecular mechanisms underlying the development of MCH neurons and possibly of other hypothalamic cell types.

## Introduction

Melanin-concentrating hormone (MCH) is a neuropeptide synthesized by hypothalamic neurons that project to numerous brain areas and the cerebral ventricle (Bittencourt et al., 1992; Conductier et al., 2013; Noble et al., 2018). MCH receptor 1 (MCHR1; the sole receptor in rodents) is also found widely in the CNS (Saito et al., 2001). Consistent with its brain-wide distribution, pharmacological or genetic modifications of MCH/MCHR1 signaling affect various physiological processes, particularly those related to energy homeostasis and reward (Diniz and Bittencourt, 2017). Recent evidence from opto/chemogenetics and *in vivo* calcium imaging experiments have highlighted the importance of MCH neuronal activity in regulating rapid eye movement (REM) sleep (Jego et al., 2013; Konadhode et al., 2013; Vetrivelan et al., 2016; Blanco-Centurion et al., 2019; Komagata et al., 2019), feeding behavior (Domingos et al., 2013; Noble et al., 2018; Dilsiz et al., 2020), and memory (Izawa et al., 2019; Kosse and Burdakov, 2019; Concetti et al., 2020).

Despite the growing body of literature on the physiology of MCH neurons, the number of studies focusing on their developmental process is limited. In rodents, the birth of MCH neurons occurs in mid-to-late gestation [embryonic day (E)10–E16 in rats and E9–E14 in mice] and the expression of MCH becomes evident in the prenatal period (Brischoux et al., 2001; Steininger et al., 2004; Croizier et al., 2010; Díaz et al., 2014). MCH neurons have at least two subpopulations, which are characterized by their neurochemical phenotypes, birthdates, and projection patterns (Brischoux et al., 2002; Cvetkovic et al., 2004; Croizier et al., 2010). One population co-expresses cocaine- and amphetamine-regulated transcript (CART) and neurokinin-3 receptor (NK3R), the other expresses neither of these molecules. The latter (i.e., MCH^+^CART^−^NK3R^−^ group) corresponds to initially generated MCH neurons, localized in the lateral hypothalamic area (LHA), and is the main source of descending MCH fibers to the spinal cord. The later born MCH^+^CART^+^NK3R^+^ population is distributed in the LHA and more medial hypothalamic areas and constitutes the major fraction of cortically projecting MCH neurons. The existence of these two subpopulations was recently corroborated by a single-cell transcriptomic analysis of MCH^+^ cells in the mouse LHA (Mickelsen et al., 2019). The heterogeneous nature of MCH neurons may contribute to parallel regulation of memory processes and muscle tone during REM sleep (Hanriot et al., 2007; Hassani et al., 2009; Izawa et al., 2019). Although previous studies have identified some transcription factors and morphogens critical for MCH neuron development (Szabó et al., 2009; Sokolowski et al., 2015; Xie et al., 2017; Seifinejad et al., 2019), little is known about how the MCH neuronal subtypes are produced in the developing hypothalamus.

*In vitro* generation of hypothalamic neurons from embryonic stem cells (ESCs) or induced pluripotent stem cells (iPSCs) can provide insights into the specification programs of those neurons. Such a differentiation system was first established for mouse ESCs (mESCs) using three-dimensional (3D) culture named SFEBq (serum-free floating culture of embryoid body-like aggregates with quick reaggregation; Wataya et al., 2008). Since then, hypothalamic neurons have been made from human ESCs and iPSCs (hESCs/hiPSCs) in SFEBq-based 3D culture (Merkle et al., 2015; Ogawa et al., 2018; Kasai et al., 2020) or two-dimensional culture (Merkle et al., 2015; Wang et al., 2015; Rajamani et al., 2018). These hESC/hiPSC cultures would be useful for basic and clinical research on the human hypothalamus, but there are two advantages of using mESCs over hESCs/hiPSCs for general purposes: a shorter differentiation period and direct comparability with *in vivo* data from animal experiments. However, to our knowledge, no previous study has focused on MCH neuron development in mESC-based differentiation systems.

Currently, SFEBq is the only established method to efficiently induce hypothalamic differentiation of mESCs. We therefore aimed to evaluate whether this 3D culture system is suitable for analyzing the developmental process of MCH neurons. To this end, we followed the original SFEBq method and induced hypothalamic-like tissues from mESCs (ES-Hypo, hereafter). We first characterized the temporal pattern of neuronal differentiation in ES-Hypo and then traced the generation of MCH neurons in this developing tissue. We also examined the similarity of mESC-derived MCH neurons to native MCH neurons by assessing their morphologic maturation, neuronal connectivity, and neuropeptide/neurotransmitter phenotype. Finally, ES-Hypo was used to demonstrate an essential role of Hedgehog (Hh) signaling in the production of the neurochemical subtypes of MCH neurons. Our data also include data obtained from the observation of the development of orexin/hypocretin neurons, a well-defined wake-promoting cell group adjacent to MCH neurons in the LHA (Soya and Sakurai, 2020).

## Materials and Methods

### Cell culture

**Figure 1.**
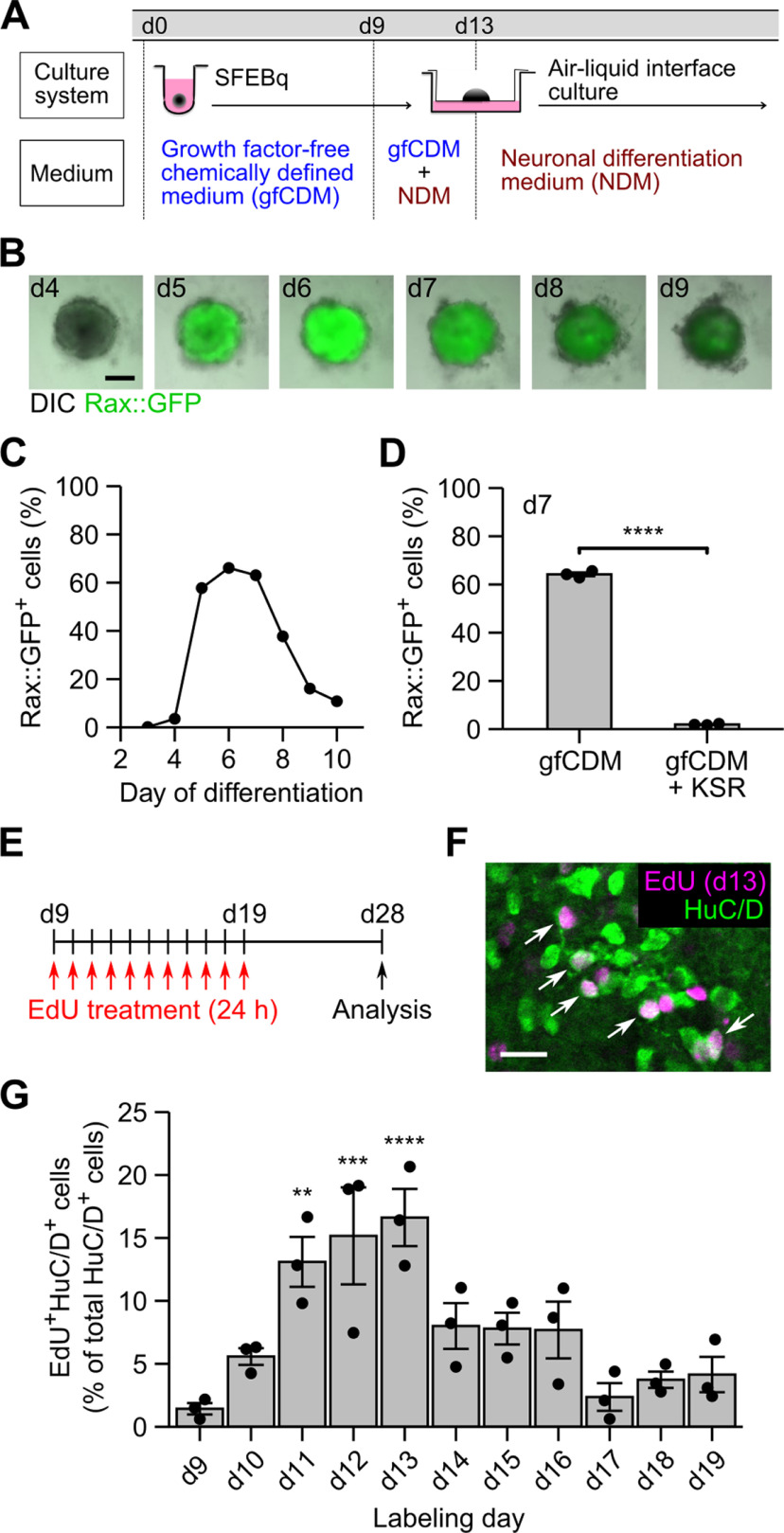
Temporal pattern of neuronal differentiation in ES-Hypo. ***A***, Culture protocol for ES-Hypo induction. d, day. ***B***, Fluorescence images of SFEBq aggregates showing the Rax::GFP expression during days 4–9. Scale bar: 200 μm. ***C***, The percentage of Rax::GFP^+^ cells during days 3–10 in a single experimental batch. ***D***, The percentage of Rax::GFP^+^ cells on day 7 in multiple experimental batches (*n *=* *3). SFEBq culturing were performed in normal differentiation medium (gfCDM) or KSR-supplemented differentiation medium (gfCDM + KSR). ****p <* *0.0001 by Welch’s *t* test. ***E***, A schematic illustration of the birth-dating analysis of postmitotic neurons in ES-Hypo. ***F***, A representative image of day-28 ES-Hypo stained for EdU and HuC/D. The cell aggregate was treated with EdU on day 13. Arrows indicate double-positive cells (i.e., postmitotic neurons born on day 13). Scale bar: 20 μm. ***G***, Summary of the birthdates of postmitotic neurons in ES-Hypo. The percentage of EdU^+^HuC/D^+^ cells among total HuC/D^+^ cells was quantified for each day of EdU labeling. *n *=* *3 aggregates per day. **p <* *0.01, ***p <* *0.001, and ****p <* *0.0001 versus day 9 by Dunnett test.

**Figure 2.**
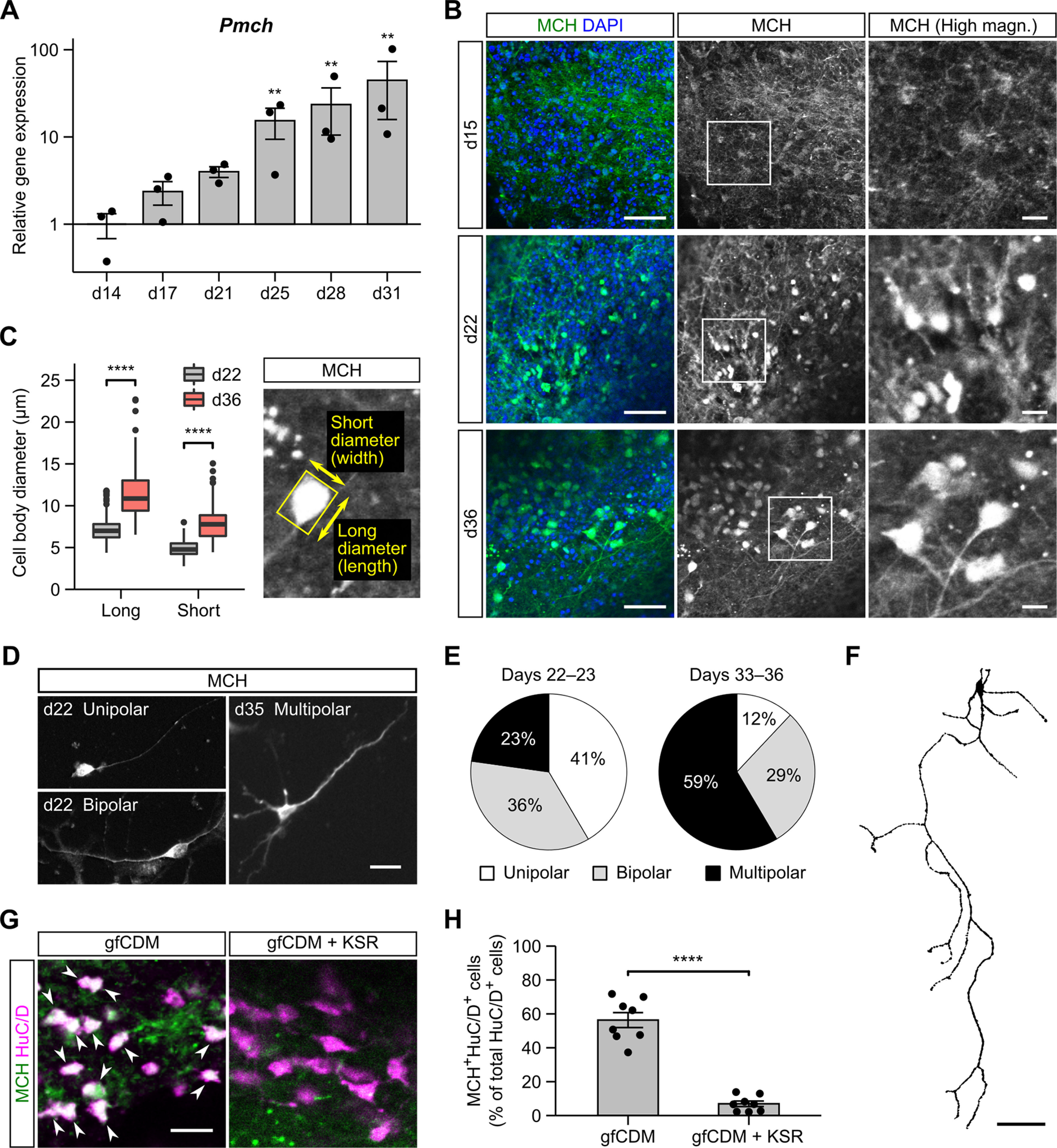
Generation of MCH neurons in ES-Hypo. ***A***, The qRT-PCR-based analysis of the *Pmch* expression during ES-Hypo differentiation. *Pmch* encodes the precursor of MCH. Data were normalized to *Actb* and gene expression on day 14 and plotted in log10 scale. *n *=* *3 experiments. **p <* *0.01 versus day 14 by Dunnett test. ***B***, Immunofluorescence staining of ES-Hypo for MCH on days 15, 22, and 36. Nuclei were stained with DAPI. The right panels show high-magnification images of the boxed regions in the middle panels. Scale bars: 50 μm (low magnification) and 10 μm (high magnification). ***C***, Quantification of the cell body diameter for MCH-ir cells on days 22 and 36. The long and short diameters for each MCH-ir cell were measured as shown in the right panel. *n *=* *300 cells per day. ****p <* *0.0001 by Welch’s *t* test. ***D–F***, The analysis of MCH-ir cells in the dissociation culture. Cells were dissociated from ES-Hypo on days 19–20 or 30–33 and cultured in a monolayer for 3 d. Representative morphologies of MCH-ir cells are shown in fluorescence images (***D***) and a cell trace (***F***). Scale bars: 20 μm (***D***) and 50 μm (***F***). The percentage of MCH-ir cells showing unipolar, bipolar, or multipolar morphology is presented in the pie charts (***E***) for days 22–23 (*n *=* *272 cells) and days 33–36 (*n *=* *200 cells). Functional properties of MCH-ir cells on day 36 were evaluated by calcium imaging as shown in Extended Data Figure 2-1. ***G***, Representative images of day-36 mESC aggregates immunostained for MCH and HuC/D. The aggregates were initially differentiated in gfCDM (left, corresponding to ES-Hypo) or gfCDM + KSR (right). Arrowheads indicate double-positive cells. Scale bar: 20 μm. ***H***, The percentage of MCH^+^HuC/D^+^ cells among total HuC/D^+^ cells under the culture conditions shown in ***G***. *n *=* *8 aggregates per condition. ****p <* *0.0001 by Welch’s *t* test.

mESCs were cultured under standard conditions (37°C, 95% air, 5% CO_2_) according to the original paper of ES-Hypo (Wataya et al., 2008), with some modifications. Two mESC lines, EB5 (RCB, #AES0151, RRID: CVCL_J648) and its Rax::GFP knock-in subclone (RCB, #AES0145, RRID: CVCL_J650), were maintained on gelatin-coated dishes in Glasgow’s minimum essential medium (Wako, #078-05525) supplemented with 1% FBS (Nichirei Biosciences, #171012), 10% knock-out serum replacement (KSR; Invitrogen, #10828028), 0.1 mm nonessential amino acids (Invitrogen, #11140050), 1 mm sodium pyruvate (Sigma, #S8636), 0.1 mm 2-mercaptoethanol (Kanto Chemical, #25099-30), 2000 U/ml leukemia inhibitory factor (Wako, #195-16053), and 20 μg/ml blasticidin S (Wako, #029-18701). For SFEBq culture, mESCs were dissociated to single cells in 0.25% Trypsin-EDTA (Invitrogen, #25200072) and quickly reaggregated in growth factor-free chemically defined medium (gfCDM; 3000 cells/100 μl/well) using low-cell-adhesion U-bottom 96-well plates (Sumilon PrimeSurface 96U plate, Sumitomo Bakelite). The gfCDM consisted of 1:1 Iscove’s modified Dulbecco’s medium (Invitrogen, #31980030)/Ham’s F12 (Invitrogen, #31765035), 1% chemically defined lipid concentrate (Invitrogen, #11905031), 450 μm monothioglycerol (Sigma, #M6145), and 5 mg/ml purified BSA (Sigma, #A9418). In some experiments (Figs. 1*D*, 2*G*,*H*), SFEBq culturing was performed in gfCDM supplemented with 10% KSR (gfCDM + KSR). The day on which mESCs were seeded for SFEBq culture was defined as differentiation day 0.

10.1523/ENEURO.0442-21.2022.f2-1 Extended Data Figure 2-1 Calcium imaging of mESC-derived MCH cells in the dissociation culture on day 36. A, Ca2+ response to high-K+ stimulation. The graph shows the time course of fluorescence signals from respective MCH cells (n = 29), which were loaded with Calbryte-520. ECF was changed from ACSF to 50 mm KCl-containing ACSF as indicated by arrow. Representative images of an MCH cell are shown on top of the graph; Calbryte-520 fluorescence at 120 s (left) and 370 s (middle), and postimmunocytochemistry (ICC) for MCH (right). B, A control experiment for high-K+ stimulation (n = 29). ECF were changed from ACSF to ACSF. C, D, Ca2+ response to glutamate (C, n = 25) or GABA (D, n = 36). The reagents were applied at final concentration of 100 μm as indicated by arrows. E, The percentage of MCH+ cells responding to high-K+, glutamate, or GABA. Download Figure 2-1, TIF file.

The original culture protocol for ES-Hypo includes fluorescence-activated cell sorting (FACS) of Rax::GFP^+^ hypothalamic progenitors on day 7 (Wataya et al., 2008). In the present study, this step was omitted because only WT mESCs were used for neuronal differentiation, except for some experiments (Fig. 7). Therefore, aggregated WT mESCs were maintained in gfCDM without any medium exchange until day 9, when 100 μl of neuronal differentiation medium (NDM) was added per well. The NDM contained DMEM/Ham’s F12 (Wako, #042-30555) supplemented with 35 mm glucose, N2 (Wako, #141-08941), MACS NeuroBrew-21 (without vitamin A; Miltenyi Biotech, #130-097-263), 10 ng/ml human CNTF (Wako, #032-18851), and penicillin/streptomycin (Wako, #168-23191). When FACS was performed on day 7, sorted Rax::GFP^+^ and GFP^−^ cells were reaggregated in DFK medium (5000 cells/100 μl/well) using low-cell-adhesion U-bottom 96-well plates and supplied with NDM on day 10 (100 μl/well). The DFK medium contained DMEM/Ham’s F12 (Wako, #048-29785) supplemented with 10% KSR, 35 mm glucose, 10 μm Y-27632 (Wako, #034-24024), and penicillin/streptomycin. From day 13, aggregates were cultivated at the air-liquid interface using Millicell cell culture inserts (Millipore, #MCSP06H48, RRID: SCR_015799) in NDM. Half the volume of the medium was changed every 2–3 d. In some experiments (Figs. 5, 6), Smoothened agonist (SAG; Cayman Chemical, #11914) was added to the differentiation media at 30 nm from day 4 onward.

For the dissociation culture of mESC-derived neurons, cells were dissociated from aggregates on days 19–20 or 30–33 using Neuron Dissociation Solutions (Wako, #297-78101) and plated onto poly-D-lysine/laminin-coated coverslips or 24-well plates at a density of 5.0–8.5 × 10^4^ cells/cm^2^ in NDM. After 3 d of adherent culture, cells were subjected to an immunocytochemical analysis or calcium imaging experiment.

### Quantification of Rax::GFP^+^ cells

To examine the time-dependent change of the Rax::GFP expression, differentiating mESC aggregates (eight aggregates per day) from a single experimental batch were dissociated with Accumax (Innovative cell technologies, #AM105) to single cells, and their fluorescence images were acquired with a DMI6000B microscope (Leica MicroSystems). Rax::GFP^+^ cells were manually quantified using the cell counter plugin for Fiji (a distribution of ImageJ, RRID: SCR_002285; Schindelin et al., 2012). To verify the reproducible induction of Rax::GFP^+^ cells, their percentage was measured by flow cytometry in multiple experimental batches on day 7.

### Flow cytometry and cell sorting

To measure the percentage of Rax::GFP^+^ cells on day 7, cells were dissociated from at least 32 aggregates per batch with Accumax and suspended in Cell Staining Buffer (BioLegend, #420201). Dead cells were stained with 7-AAD (Tonbo Biosciences, #13-6993), and the 7-AAD^−^ live cell fraction (>10,000 events) was analyzed using a BD FACS Calibur (BD Biosciences) and the BD CellQuest Pro software program (BD Biosciences, RRID: SCR_014489). WT mESC-derived cells were used as a negative control for setting the Rax::GFP^+^ gate.

For FACS sorting, day-7 aggregates were pretreated with 10 μm Y-27632 for 1 h to minimize cell death during the experiment. Accumax-dissociated cells were suspended in FACS buffer consisting of HBSS(−) (Wako, #085-09355), 0.5% BSA, 1 mm EDTA, and 10 μm Y-27632. Dead cells were stained with propidium iodide (PI; Sigma, #P4170), and Rax::GFP^+^ and GFP^−^ cells were sorted from the PI^−^ live cell fraction using MoFlo Astrios (Beckman Coulter). Sorted cells were collected in ice-cold DFK medium.

### qRT-PCR

Total RNA was collected from differentiating mESC aggregates (8 aggregates per sample) using a FastPure RNA kit (TaKaRa, #9190) and converted into cDNA with Superscript II reverse transcriptase (Invitrogen, #18064022). qPCR was performed using THUNDERBIRD SYBR qPCR Mix (Toyobo, #QPS-201) and the ABI PRISM 7900HT system (Applied Biosystems). Data were normalized to the expression of the β-actin gene (*Actb*). The following primers were used: *Actb*, forward 5′-CTAAGGCCAACCGTGAAAAG-3′, reverse 5′-ACCAGAGGCATACAGGGACA-3′; *Pmch*, forward 5′-CACAGGAAAAGAGAGAAATTGGG-3′, reverse 5′-TGTAAGGATGTTGCGGACC-3′; *Hcrt*, forward 5′-TCTTGGGTATTTGGACCACTG-3′, reverse 5′-CCCAGGGAACCTTTGTAGAAG-3′.

### Calcium imaging

Dissociation cultures prepared in 24-well plates were used for calcium imaging on day 36. Before imaging, cells were subjected to a loading of the Ca^2+^ indicator Calbryte-520 AM (AAT Bioquest, #20650) and nuclear staining with Hoechst 33 342 (Dojindo, #H342) as follows. 2× loading solution containing 10 μm Calbryte-520 AM, 5 μg/ml Hoechst 33342, and 0.04% Pluronic F-127 (Biotium, #59004) was prepared in artificial CSF (ACSF). The composition of ACSF was (in mm) 132 NaCl, 3 KCl, 1.3 MgCl_2_, 2.4 CaCl_2_, 20 NaHCO_3_, 1.2 KH_2_PO_4_, 3 HEPES, and 10 glucose. Half the volume of the culture medium was changed with 2× loading solution, and cells were cultured for 60–90 min. Cells were then washed and maintained in ACSF at room temperature (RT) before and during imaging. To measure Ca^2+^ responses, time-lapse images of cells were captured at six time points (0, 60, 120, 250, 310, and 370 s) using the Opera Phenix high-content imaging system (PerkinElmer). Cells were stimulated with KCl, glutamate, or GABA at 210 s. For KCl stimulation, extracellular fluid (ECF) was manually changed from ACSF to high-K^+^ ACSF containing 50 mm KCl and 85 mm NaCl. Glutamate and GABA were applied by replacing half the volume of ECF with ACSF containing 200 μm glutamate or GABA. After calcium imaging, cells were fixed with 4% PFA in PBS for 10 min and subjected to immunocytochemistry for MCH to identify MCH-expressing cells. Calbryte-520 fluorescence from the identified MCH^+^ cells was quantified using the Harmony software program (PerkinElmer) by measuring mean pixel intensity in somatic areas, which were automatically detected based on nuclear staining with Hoechst. The fluorescence intensity was graphed as Δ*F/F_0_*, where Δ*F* is the intensity difference compared with background fluorescence (*F_0_*). The intensity value at 120 s was used as *F_0_*. Responding cells were defined as the cells with Δ*F/F_0_* > 0.5 at 370 s because spontaneous fluctuations in Δ*F/F_0_* were limited in ±0.5.

### Immunofluorescence staining

After more than two weeks of differentiation, mESC aggregates were analyzed by whole-mount immunohistochemistry as follows. First, aggregates were fixed with 4% PFA in PBS for 20 min at RT. Fixed aggregates were blocked and permeabilized with 5% normal donkey serum (NDS) and 0.5% Triton X-100 in PBS for 1 h at RT and then incubated with primary antibodies overnight at 4°C, except that incubation with an anti-orexin-A antibody was performed for 36–48 h. After washing in PBS supplemented with 0.05% Triton X-100 (PBSX), aggregates were incubated with appropriate secondary antibodies for 1 h at RT. In this step, nuclear staining with DAPI (Dojindo, #D523) was performed simultaneously. Antibodies and DAPI were diluted with PBS containing 5% NDS and 0.1% Triton X-100 just before use. Finally, aggregates were washed in PBSX and mounted on glass slides using Fluoromount (Diagnostic BioSystems, #K024). The antibodies used in this study are listed in Table 1.

**Table 1:** Primary and secondary antibodies used for immunofluorescence staining

	Host	Dilution	Source; catalog number	RRID
Primary antibody				
HuC/D	Mouse	1 μg/ml	Thermo Fisher Scientific; A-21271	AB_221448
MCH	Rabbit	1:3000	Phoenix Pharmaceuticals; H-070-47	AB_10013632
Orexin-A	Goat	1:250	Santa Cruz Biotechnology; sc-8070	AB_653610
GAD67	Mouse	1:500	Millipore; MAB5406	AB_2278725
VGLUT2	Guinea pig	1:500	Millipore; AB2251-I	AB_2665454
Nesfatin-1	Sheep	2 μg/ml	R&D Systems; AF6895	AB_10972964
CART	Chicken	1:500	Millipore; AB5340P	AB_91795
Rax	Guinea pig	1:2000	TaKaRa; M229	AB_2783559
Pax6	Mouse	1:100	DSHB; PAX6	AB_528427
Nkx2.1	Rabbit	1:200	Santa Cruz Biotechnology; sc-13040	AB_793532
Nkx2.1	Mouse	1:500	MBL; K0121-3	AB_592930
Sox1	Goat	1:500	R&D Systems; AF3369	AB_2239879
Foxg1	Rabbit	1:1000	TaKaRa; M227	AB_2827749
Nkx2.2	Mouse	1:50	DSHB; 74.5A5	AB_531794
NK3R	Rabbit	1:1000	Novus Biologicals; NB300-102SS	AB_2287128
GFP	Chicken	1:500	Thermo Fisher Scientific; A10262	AB_2534023
Secondary antibody				
Anti-mouse, Alexa Fluor 488	Goat	1:1000	Thermo Fisher Scientific; A-11029	AB_2534088
Anti-rabbit, Alexa Fluor 488	Donkey	1:1000	Thermo Fisher Scientific; A-21206	AB_2535792
Anti-chicken, Alexa Fluor 488	Donkey	1:500	Jackson ImmunoResearch; 703-545-155	AB_2340375
Anti-goat, Alexa Fluor 488	Donkey	1:1000	Thermo Fisher Scientific; A-11055	AB_2534102
Anti-mouse, Alexa Fluor 555	Donkey	1:1000	Thermo Fisher Scientific; A-31570	AB_2536180
Anti-rabbit, Alexa Fluor 555	Donkey	1:1000	Thermo Fisher Scientific; A-31572	AB_162543
Anti-goat, Alexa Fluor 555	Donkey	1:1000	Thermo Fisher Scientific; A-21432	AB_2535853
Anti-sheep, Alexa Fluor 555	Donkey	1:1000	Thermo Fisher Scientific; A-21436	AB_2535857
Anti-mouse, Alexa Fluor 594	Donkey	1:1000	Thermo Fisher Scientific; A-21203	AB_141633
Anti-rabbit, Alexa Fluor 594	Donkey	1:1000	Thermo Fisher Scientific; A-21207	AB_141637
Anti-guinea pig, Cy3	Donkey	1:500	Jackson ImmunoResearch; 706-165-148	AB_2340460
Anti-mouse, Alexa Fluor 647	Donkey	1:1000	Thermo Fisher Scientific; A-31571	AB_162542
Anti-rabbit, Alexa Fluor 647	Donkey	1:1000	Thermo Fisher Scientific; A-31573	AB_2536183

For the immunohistochemical analysis of aggregates cultured for 7–13 d, they were fixed with 4% PFA for 1 h at RT and cryoprotected in sucrose solutions of increasing concentrations (10, 20, and 30%) for 30 min each, followed by embedding in O.C.T. compound (Sakura, #4583). Serial 10-μm-thick cryosections were prepared with a CM3050S cryostat (Leica Biosystems) and mounted on PLATINUM PRO adhesive slides (Matsunami, #SPRO-02). The sections were blocked and permeabilized with 5% NDS and 0.1% Triton X-100 in PBS for 30 min at RT and then immunostained as described above.

For the immunocytochemical analysis of dissociation cultures, cells were fixed with 4% PFA for 10 min at RT and then processed in the same way as cryosections.

Fluorescence images of whole-mount aggregates were obtained using a LSM710 confocal microscope (Carl Zeiss), and those of cryosections and dissociation cultures were obtained using a DMI6000B microscope, LSM710 confocal microscope, or BZ-9000 microscope (Keyence).

### Birth-dating analysis of postmitotic neurons

During days 9–19, mESC aggregates were cultured for 24 h in the differentiation medium containing 10 μm 5-ethynyl-2’-deoxyuridine (EdU) by the day. After the EdU treatment, aggregates were washed and cultured in differentiation medium free of EdU until day 28 and fixed with 4% PFA for 20 min at RT. Aggregates were then subjected to visualization of EdU, followed by immunofluorescence staining for the neuronal marker HuC/D. The EdU labeling and staining were performed using a Click-iT EdU Alexa Fluor 647 Imaging kit (Invitrogen, #C10340). Fluorescence images were captured with a LSM710 confocal microscope, and EdU^+^HuC/D^+^ double-positive cells were considered to be postmitotic neurons born during EdU treatment. Cell counts were performed as described below.

### The imaging analysis of immunofluorescence data

The long and short diameters of MCH^+^ cell bodies were measured using the straight line and measure tools in Fiji. In the birth-dating analysis of postmitotic neurons, the percentage of EdU^+^ cells in the HuC/D^+^ neuronal population was determined as follows. First, in the fluorescence images acquired from each aggregate, HuC/D^+^ cells were manually quantified using the cell counter plugin for Fiji (200–500 positive cells per aggregate). Next, EdU^+^ cells were counted in the prequantified HuC/D^+^ population to calculate the percentage of its EdU^+^ fraction. Although a part of HuC/D^+^ cells exhibited diffuse EdU staining, they were also considered as EdU^+^ cells. This analysis protocol was also applied to determine the MCH^+^ percentage in the HuC/D^+^ population, the HuC/D^+^ percentage in the MCH^+^ population, and the positive rates of neurochemical markers in the MCH^+^ population. For the analysis of cryosections, fluorescence images were acquired from three non-adjacent sections per aggregate. Several images from day-7 aggregates were analyzed using the Columbus software program (PerkinElmer) to calculate the Pax6^+^ or Nkx2.1^+^ percentage in the Rax^+^ cell population semi-automatically (200–400 Rax^+^ cells were detected per aggregate).

### Statistical analysis

Statistical analyses were performed using the R software program (version 4.0.5, The R Foundation, RRID: SCR_001905). Results of grouped data were presented as the mean ± SEM in the text or figures (bar plots). Some data were presented with box plots showing the median (bold line), interquartile range (IQR; box), and ±1.5 IQR (whisker) with outliers (Fig. 2*C*) or all data points (Fig. 5*H*). Statistical significance between two groups was assessed by Welch’s *t* test, except for Figure 5*H*, in which two groups were compared using the Brunner–Munzel test, which does not require assumptions of normality and homoscedasticity. For time series data, statistical significance between the earliest time point and other timepoints was assessed by Dunnett test. For statistical analysis of qRT-PCR data, a log10 transformation was performed to reduce variability of data. The results of the statistical analyses are summarized in Table 2.

**Table 2:** Statistical table

Figure	Group comparison	Data structure	Type of test	*p* value	95% confidence interval
1*D*	gfCDM vs gfCDM + KSR	Normal distribution	Welch’s *t* test	*p *<* *0.0001	59.03, 65.52
1*G*	d9 vs d10	Normal distribution	Dunnett test	*p *=* *0.55798	−3.63, 11.94
	d9 vs d11	Normal distribution	Dunnett test	*p *=* *0.00166	3.89, 19.46
	d9 vs d12	Normal distribution	Dunnett test	*p *=* *0.00033	5.95, 21.52
	d9 vs d13	Normal distribution	Dunnett test	*p *<* *0.0001	7.41, 22.98
	d9 vs d14	Normal distribution	Dunnett test	*p *=* *0.12627	−1.21, 14.37
	d9 vs d15	Normal distribution	Dunnett test	*p *=* *0.14666	−1.42, 14.16
	d9 vs d16	Normal distribution	Dunnett test	*p *=* *0.15859	−1.53, 14.05
	d9 vs d17	Normal distribution	Dunnett test	*p *=* *0.99996	−6.85, 8.73
	d9 vs d18	Normal distribution	Dunnett test	*p *=* *0.95885	−5.48, 10.09
	d9 vs d19	Normal distribution	Dunnett test	*p *=* *0.90193	−5.06, 10.51
2*A* ^*^	d14 vs d17	Normal distribution	Dunnett test	*p *=* *0.5707	−0.20, 1.51
	d14 vs d21	Normal distribution	Dunnett test	*p *=* *0.1578	−5.06, 10.51
	d14 vs d25	Normal distribution	Dunnett test	*p *=* *0.0093	0.28, 1.99
	d14 vs d28	Normal distribution	Dunnett test	*p *=* *0.0036	0.45, 2.16
	d14 vs d31	Normal distribution	Dunnett test	*p *=* *0.0010	0.66, 2.38
2*C*, “long”	d22 vs d36	Normal distribution	Welch’s *t* test	*p *<* *0.0001	−4.53, −3.86
2*C*, “short”	d22 vs d36	Normal distribution	Welch’s *t* test	*p *<* *0.0001	−3.15, −2.69
2*H*	gfCDM vs gfCDM + KSR	Normal distribution	Welch’s *t* test	*p *<* *0.0001	38.75, 60.04
3*B* ^*^	d14 vs d31	Normal distribution	Welch’s *t* test	*p *=* *0.01818	−2.89, −0.52
5*C*, top	SAG (−) vs (+)	Normal distribution	Welch’s *t* test	*p *<* *0.0001	70.25, 93.41
5*C*, bottom	SAG (−) vs (+)	Normal distribution	Welch’s *t* test	*p *<* *0.0001	−92.79, −82.23
5*F*	SAG (−) vs (+)	Normal distribution	Welch’s *t* test	*p *=* *0.009963	−45.36, −8.52
5*H*	SAG (−) vs (+)	Normality not assumed	Brunner–Munzel test	*p *<* *0.0001	0.77, 1.04

* Log10-transformed data were used for statistical analysis.

## Results

### ES-Hypo recapitulates the temporal pattern of hypothalamic development

The SFEBq method, using gfCDM, efficiently induces Rax^+^ hypothalamic progenitors from mESCs after 7 d of differentiation (Wataya et al., 2008). Rax is a transcription factor specifically expressed in the hypothalamic and retinal neuroepithelium (Furukawa et al., 1997; Mathers et al., 1997; Shimogori et al., 2010; Lu et al., 2013). The Rax^+^ progenitors subsequently generate their neuronal derivatives, such as vasopressin cells, after 20 or more days (Wataya et al., 2008). In this culture system, however, the time course of neuronal differentiation has not been fully characterized. We therefore started our study by systematically tracing Rax^+^ cell induction and subsequent neurogenesis during ES-Hypo development (Fig. 1*A*).

To monitor Rax^+^ cell generation in real time, a Rax::GFP reporter mESC line (Wataya et al., 2008) was subjected to SFEBq culture. The expression of Rax::GFP was sharply increased on day 5 and was maintained until day 7 in a large proportion of aggregated cells, followed by a gradual decrease (Fig. 1*B*,*C*). A flow cytometric analysis on day 7 confirmed that Rax::GFP^+^ progenitors were reproducibly induced in gfCDM (∼64% of total cells) but greatly decreased when the medium was supplemented with KSR (∼2% of total cells; Fig. 1*D*). KSR is a widely used serum replacement with minimal growth factors, but it is reported to severely inhibit hypothalamic differentiation of mESCs via activating the insulin/PI3K/Akt pathway and rather induce telencephalic differentiation (Wataya et al., 2008). Since the expression of Rax::GFP was downregulated from days 7–10 (Fig. 1*C*), we expected that neurogenesis starts in this period. To evaluate the time course of neurogenesis, we conducted a birth-dating analysis using the thymidine analog EdU during days 9–19. Newly born postmitotic neurons were labeled with EdU by the day, and they were quantified on day 28 as EdU and HuC/D (a neuronal marker) double-positive cells (Fig. 1*E*,*F*). The results of the analysis were summarized in Figure 1*G*. The birth of postmitotic neurons has already occurred on day 9, and it reached a peak during days 11–13 (roughly half of HuC/D^+^ neurons were born in this period) and persisted at lower levels thereafter. Eventually, 85% of HuC/D^+^ neurons were generated throughout the labeling period. Taken together, hypothalamic progenitor specification occurs within the first week of mESC differentiation and after neurogenesis, mostly within the second and third weeks. Considering that mESCs are derived from blastocysts (∼E3.5 embryos), this time course resembles the temporal pattern of embryonic development of the mouse hypothalamus (Shimada and Nakamura, 1973; Ishii and Bouret, 2012; Lu et al., 2013).

### Development of MCH neurons in ES-Hypo

Based on the determined time course of neurogenesis, we explored MCH neuron development in ES-Hypo after two weeks of differentiation. qRT-PCR showed that MCH mRNA levels increased exponentially during weeks 3–5 (Fig. 2*A*). MCH-immunoreactivity was faintly observed on day 15, but clear MCH-immunoreactive (ir) cell bodies and fibers appeared on day 22 (Fig. 2*B*). These MCH-ir cell bodies were characterized by round shapes and small diameters (<10 μm), both of which are features of immature MCH neurons (Steininger et al., 2004; Li et al., 2018). After 36 d of culture, MCH-ir cells were often angular-shaped and larger in comparison to MCH-ir cells on day 22 (Fig. 2*B*,*C*), which is reminiscent of neuronal maturation *in vivo* (Steininger et al., 2004; Li et al., 2018). Another hallmark of neuronal maturation is neurite growth; while young MCH neurons have only one or two neurites (i.e., unipolar or bipolar cells), a major fraction of adult MCH neurons are multipolar cells with an axon and 2–5 primary dendrites (Steininger et al., 2004; Diniz et al., 2019). To assess morphologic changes in mESC-derived MCH^+^ cells in terms of neurite number, we prepared dissociation cultures from ES-Hypo on different days. Immunocytochemistry showed that 77% of MCH-ir cells were classified into unipolar or bipolar cells on days 22–23, but 59% of MCH-ir cells exhibited a multipolar morphology on days 33–36 (Fig. 2*D*,*E*). Some of the multipolar cells displayed an extended and branched axonal process (Fig. 2*F*). We also performed a calcium imaging experiment in the dissociation culture on day 36 and found that most MCH-ir cells were activated by KCl or glutamate but not by GABA (Extended Data Fig. 2-1). These morphologic and functional properties strongly support the neuronal identity of MCH-ir cells, and we further confirmed that the neuronal marker HuC/D was expressed in 93.2 ± 1.2% of MCH-ir cells (*n *=* *8 aggregates on day 36; Fig. 2*G*, left). The MCH^+^ percentage of total HuC/D^+^ neurons varied among individual aggregates (37–72%) but became much lower (2–13%) when SFEBq culturing had been performed in KSR-containing medium (Fig. 2*G*,*H*). These data indicate that ES-Hypo robustly generates MCH neurons, which reproduce the morphologic development of native MCH neurons.

### Putative synaptic connections between MCH and orexin neurons in ES-Hypo

**Figure 3.**
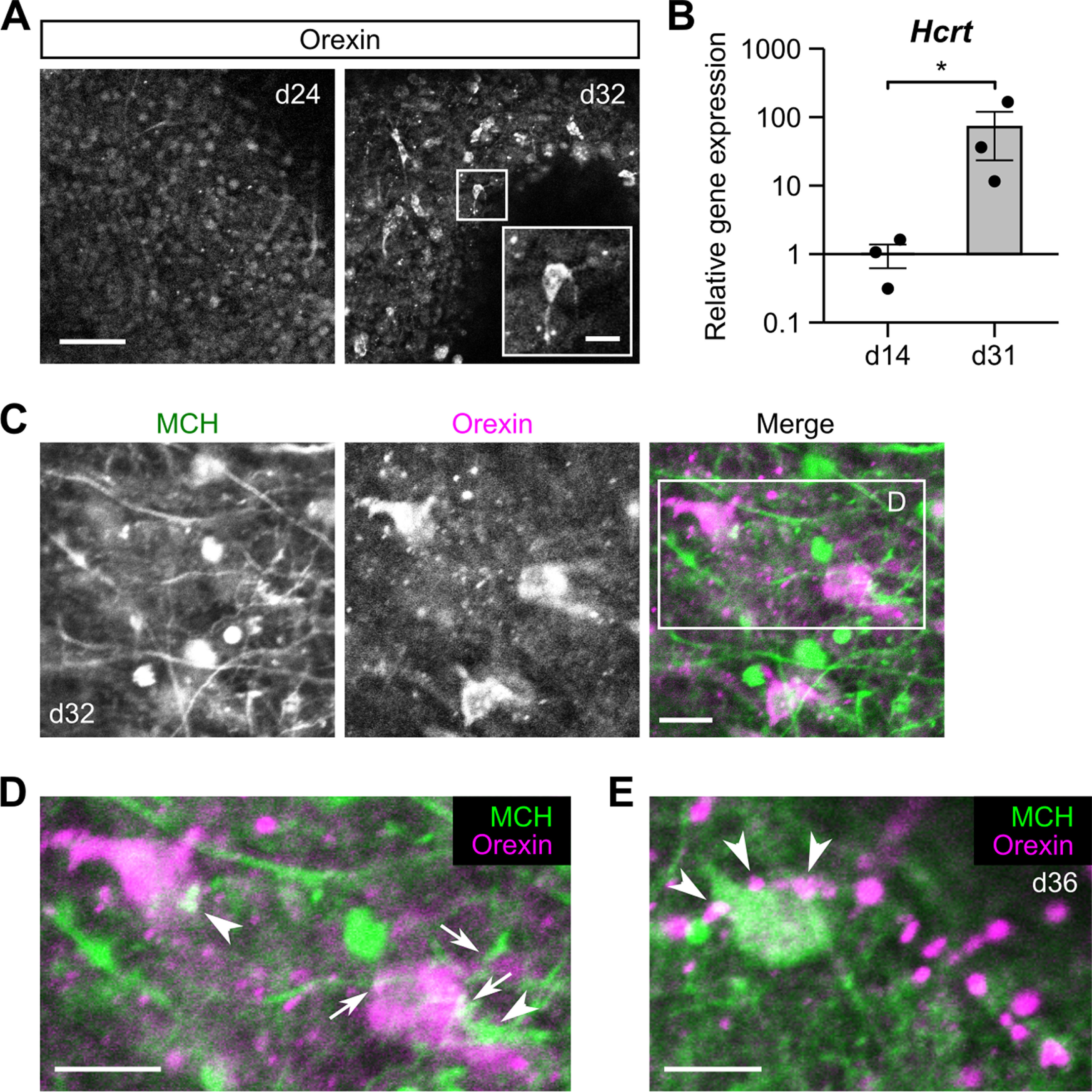
Reciprocal connectivity between MCH and orexin neurons in ES-Hypo. ***A***, Representative images of ES-Hypo immunostaind for orexin on days 24 and 32. The inset shows a magnified view of an orexin-ir cell in the boxed region. Scale bars: 50 and 10 μm (inset). ***B***, The qRT-PCR-based analysis of the *Hcrt* expression on days 14 and 31. *Hcrt* encodes the precursor of orexin. Data were normalized to *Actb* and gene expression on day 14 and plotted in log10 scale. *n *=* *3 experiments. *p <* *0.05 by Welch’s *t* test. ***C–E***, Double-immunofluorescence images showing putative connections between MCH-ir and orexin-ir cells in ES-Hypo on days 32 and 36. The boxed region in ***C*** is magnified in ***D***. Two orexin-ir cells are contacted by MCH-ir fibers (***D***, arrows) or boutons (***D***, arrowheads). Similarly, an MCH-ir cell is closely apposed by orexin-ir boutons (***E***, arrowheads). Scale bars: 10 μm.

The axon growth observed for MCH neurons in ES-Hypo suggests that they may form synaptic connections with other differentiated neurons. There is some histologic and electrophysiological evidence to support reciprocal connections between MCH neurons and neighboring orexin neurons in the LHA (Bayer et al., 2002; Guan et al., 2002; van den Pol et al., 2004; Torterolo et al., 2006; Rao et al., 2008; Apergis-Schoute et al., 2015), which may be implicated in sleep/wake control (Konadhode et al., 2014; Hung et al., 2020). To test the possibility that such local wiring occurs in ES-Hypo, we first analyzed the differentiation of orexin neurons. Although orexin-ir cells were rarely observed before day 25, we encountered clusters of orexin-ir cells, showing neuronal morphologies, in a fraction of mESC aggregates cultured for four to five weeks (∼25% of the aggregates tested; Fig. 3*A*). Consistent with this immunoreactivity, orexin mRNA levels were significantly higher on day 31 than on day 14 (Fig. 3*B*). Based on these results, we next performed double immunofluorescence staining for MCH and orexin in ES-Hypo cultured for more than one month. Confocal microscopy of orexin-rich aggregates showed that orexin-ir cells were largely immunonegative for MCH and <10% of them were detected as double-positive cells (Fig. 3*C*). However, single-positive orexin-ir perikarya were often apposed by MCH-ir fibers or boutons (Fig. 3*D*). Similarly, we identified MCH-ir cells in contact with orexin-ir boutons (Fig. 3*E*). These findings support the hypothesis that ES-Hypo can produce some MCH-orexin neuronal networks that possibly resemble those in the LHA.

### Neuropeptide/neurotransmitter phenotype of MCH neurons in ES-Hypo

**Figure 4.**
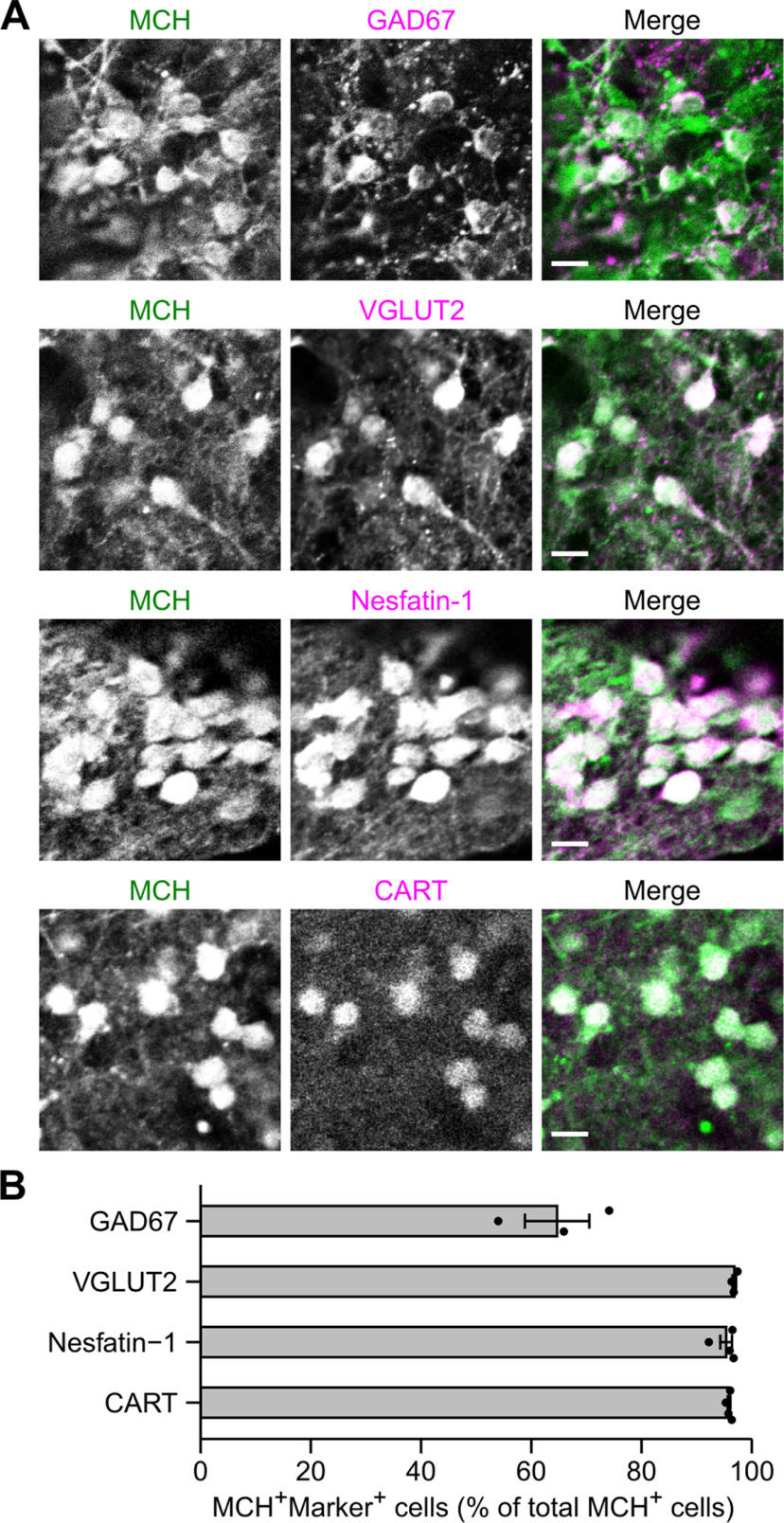
Characterization of neuropeptide/neurotransmitter phenotype of MCH neurons in ES-Hypo. ***A***, Representative images of day-36 ES-Hypo immunostained for MCH and different neurochemical markers GAD67, VGLUT2, nesfatin-1, and CART. Scale bars: 10 μm. ***B***, The percentage of MCH^+^ cells expressing different neurochemical markers. *n *=* *3–4 aggregates per marker.

MCH neurons are known to express other neuropeptides, including CART and nesfatin-1, and GABAergic/glutamatergic markers such as GAD67 (a GABA-synthesizing enzyme) and VGLUT2 (a machinery for glutamate uptake into synaptic vesicles; Broberger, 1999; Vrang et al., 1999; Brailoiu et al., 2007; Sapin et al., 2010; Chee et al., 2015; Mickelsen et al., 2017). To examine whether mESC-derived MCH neurons have a similar neurochemical profile, we performed double immunofluorescence staining for MCH and the above markers in ES-Hypo on day 36 (Fig. 4*A*). Nesfatin-1 and VGLUT2 were expressed in 95.3% and 96.8% of MCH neurons in ES-Hypo, respectively (Fig. 4*B*), which is comparable to the values for MCH neurons *in vivo* (Foo et al., 2008; Fort et al., 2008; Vas et al., 2013; Chee et al., 2015; Mickelsen et al., 2017; Schneeberger et al., 2018; Naganuma et al., 2019; Hung et al., 2020). The expression of GAD67 was previously detected in 85% of native MCH neurons by an *in situ* hybridization (ISH) analysis (Sapin et al., 2010; Jego et al., 2013; Mickelsen et al., 2017), but the GAD67-ir fraction of MCH neurons in ES-Hypo was somewhat smaller (64.7%; Fig. 4*B*). Because immunostaining methods are less sensitive than ISH in the detection of cell bodies of neurons that express low levels of GAD mRNAs (Esclapez et al., 1994), it is likely that the GAD67^+^ ratio of mESC-derived MCH neurons is similar to that of native MCH neurons. In contrast, this similarity in the population ratio was not observed for CART; while MCH^+^CART^+^ neurons constitute only half of the whole MCH^+^ population in the mouse hypothalamus (Croizier et al., 2010; Mickelsen et al., 2017), 95.9% of MCH-ir cells in ES-Hypo were labeled by an anti-CART antibody (Fig. 4*B*). Collectively, mESC-derived MCH neurons share a series of neuropeptide/neurotransmitter markers with native MCH neurons and mostly represent the CART^+^ subpopulation.

### Hh signaling is critical for generating CART-negative MCH neurons and orexin neurons from mESCs

**Figure 5.**
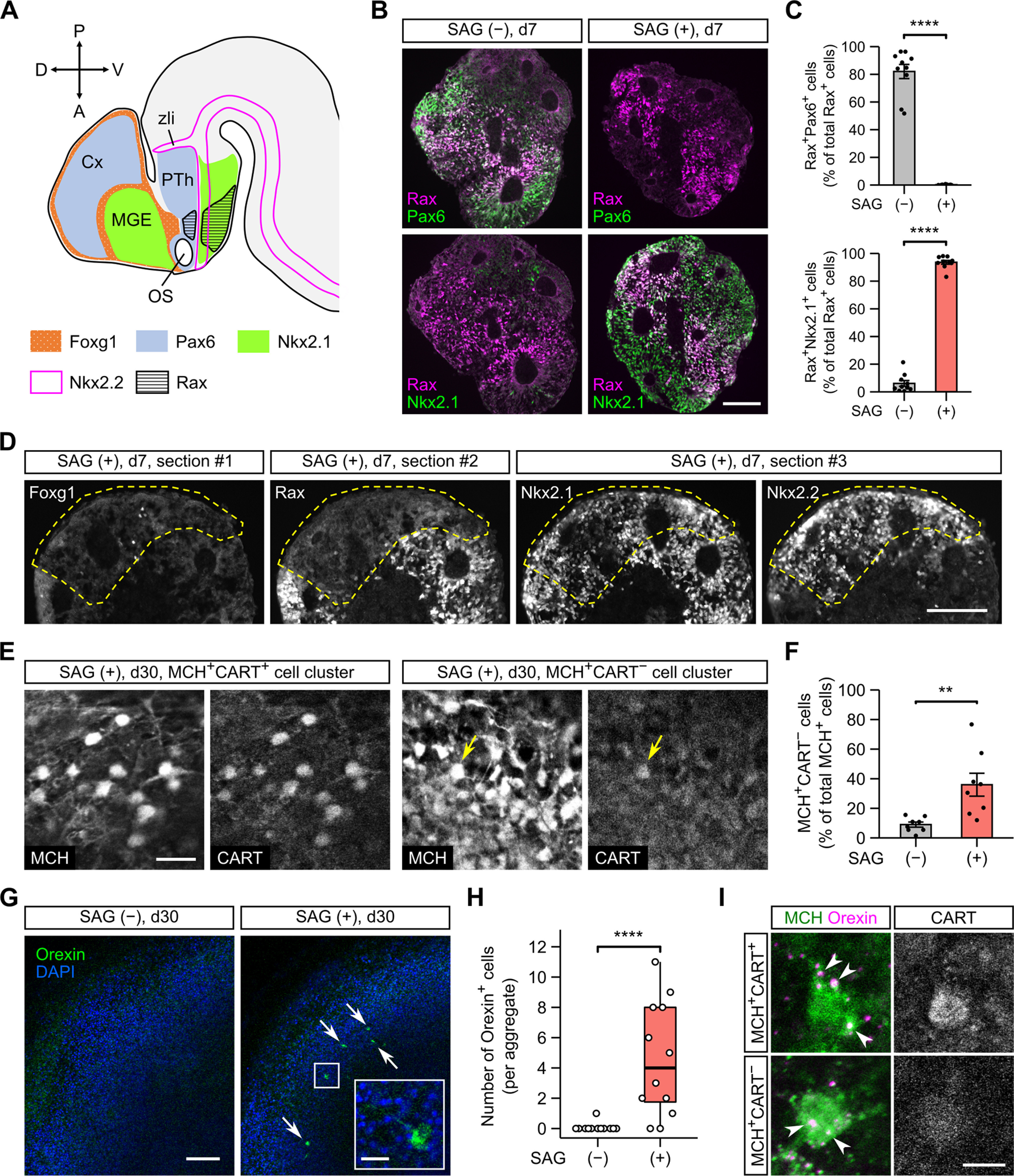
The generation of CART-negative MCH neurons and orexin neurons is increased by the activation of Hh signaling in ES-Hypo. ***A***, The regional expression of transcription factors in the telencephalon and anterior diencephalon of embryonic mouse brain around E12. The indicated expression patterns are based on the published literature (Shimamura et al., 1995; Marín et al., 2002; Shimogori et al., 2010; Lu et al., 2013; Díaz et al., 2014; Ferran et al., 2015). Cx, cortex; MGE, medial ganglionic eminence; OS, optic stalk; PTh, prethalamus; zli, zona limitans intrathalamica. ***B***, Representative images of day-7 mESC aggregates immunostained for Rax and Pax6 (upper panels) or Nkx2.1 (lower panels). The aggregates were differentiated in the absence (−) or presence (+) of 30 nm SAG. Scale bar: 100 μm. Immunostaining for the general neural progenitor marker Sox1 is presented in Extended Data Figure 5-1. ***C***, The percentage of Rax^+^ cells expressing Pax6 (top) or Nkx2.1 (bottom) on day 7 under SAG (−) and (+) conditions. *n *=* *10 aggregates per condition. ****p <* *0.0001 by Welch’s *t* test. ***D***, Serial sections from a day-7 aggregate cultured with SAG. The sections were stained for Foxg1 (#1), Rax (#2), or Nkx2.1/Nkx2.2 (#3). A Rax^−^ region is surrounded by dashed lines. Scale bar: 100 μm. ***E***, Representative immunofluorescence images of MCH^+^CART^+^ (left) and MCH^+^CART^−^ (right) cell clusters in SAG-treated aggregates on day 30. In the MCH^+^CART^−^ cluster, only one MCH^+^ cell is weakly stained for CART (arrow). Scale bar: 20 μm. In Extended Data Figure 5-2, we assessed the co-expression of MCH and HuC/D in SAG-treated aggregates. In Extended Data Figure 5-3, we assessed the co-expression of CART and NK3R in SAG-treated aggregates. ***F***, The percentage of MCH^+^CART^−^ cells among total MCH^+^ cells on day 30 under SAG (−) and (+) conditions. *n *=* *8 aggregates per condition. **p <* *0.01 by Welch’s *t* test. ***G***, Representative images of SAG-treated (right) and untreated (left) aggregates immunostained for orexin on day 30. Arrows indicate orexin-ir cells. The inset shows a magnified view of an orexin-ir cell in the boxed region. Scale bars: 100 and 20 μm (inset). ***H***, Quantification of orexin^+^ cells on day 30 under SAG (−) and (+) conditions. *n *=* *12 aggregates per condition. ****p <* *0.0001 by Brunner–Munzel test. ***I***, Representative images of an MCH^+^CART^+^ (top) or MCH^+^CART^−^ (bottom) cell, which is contacted by orexin-ir boutons (arrowheads). Triple immunostaining was performed in SAG-treated aggregates on day 36. Scale bar: 10 μm.

In the above-mentioned experiments (Figs. 1-4), SFEBq culturing was performed without exogenous morphogenic factors. This condition is reported to induce dorsal hypothalamic progenitors (Rax^+^Pax6^+^; Fig. 5*A*), whereas the addition of Shh, an endogenous Hh pathway agonist, can generate ventral hypothalamic progenitors (Rax^+^Nkx2.1^+^; Fig. 5*A*; Wataya et al., 2008). To examine whether Hh signaling affects mESC differentiation into CART-negative MCH neurons, we used SAG, a small-molecule agonist of the Hh pathway. In the absence of SAG, induced Rax^+^ progenitors were largely positive for the dorsal hypothalamic marker Pax6 but only partially positive for the ventral hypothalamic marker Nkx2.1 (Fig. 5*B*,*C*), as reported previously (Wataya et al., 2008). Treatment with SAG from day 4 did not greatly alter the Rax::GFP^+^ percentage on day 7 (49.3 ± 0.1%, *n *=* *3 experiments) but dramatically increased the Rax^+^Nkx2.1^+^ population at the cost of the Rax^+^Pax6^+^ population (Fig. 5*B*,*C*). The SAG treatment did not affect the efficiency of neural differentiation, as indicated by the expression of the general neural progenitor marker Sox1 (Extended Data Fig. 5-1). We also found that not only Rax^+^ but also Rax^−^ cells largely expressed Nkx2.1 in the SAG-treated aggregates (Fig. 5*B*). Although Nkx2.1 can be expressed in telencephalic progenitors (Shimamura et al., 1995; Marín et al., 2002; Fig. 5*A*), the pan-telencephalic marker Foxg1 was almost absent in the SAG-treated aggregates (Fig. 5*D*), suggesting that the Rax^−^Nkx2.1^+^ cells are of hypothalamic lineage. In the early hypothalamic neuroepithelium, the Rax^−^Nkx2.1^+^ territories are located anterior and posterior to the Rax^+^Nkx2.1^+^ tuberal subregion and partly overlap with a longitudinal band expressing the transcription factor Nkx2.2 (Shimamura et al., 1995; Shimogori et al., 2010; Lu et al., 2013; Díaz et al., 2014; Ferran et al., 2015; Fig. 5*A*). We confirmed that the Rax^−^Nkx2.1^+^ portions of the SAG-treated aggregates significantly overlapped with the Nkx2.2^+^ portions (Fig. 5*D*). These data indicate that the SAG treatment specifies Nkx2.1^+^ ventral hypothalamic progenitors, which comprise heterogeneous subpopulations.

Subsequent culture with the continuous addition of SAG still produced many MCH-ir neurons (Extended Data Fig. 5-2), but they contained a larger proportion of CART-negative cells in comparison to MCH neurons without SAG (Fig. 5*F*). MCH^+^CART^−^ neurons were generally found in clusters and not co-distributed with MCH^+^CART^+^ neurons (Fig. 5*E*), implying separate developmental pathways for both populations. Since CART^+^ but not CART^−^ MCH neurons specifically express NK3R *in vivo* (Croizier et al., 2010), we further tested NK3R-immunoreactivity in SAG-treated samples. Because the expression of CART was limited to the MCH^+^ cell population (Fig. 5*E*, left), we simply performed double immunostaining for CART/NK3R and found over 95% co-localization of these markers (Extended Data Fig. 5-3). This result indicates that NK3R is expressed in the MCH^+^CART^+^ but not MCH^+^CART^−^ cell group in ES-Hypo, as in the native hypothalamus. The SAG treatment also influenced the differentiation of orexin neurons. Orexin-ir cells were nearly absent in SAG-free ES-Hypo on day 30, but several orexin-ir cells were found in the majority of SAG-treated ES-Hypo on the same day (Fig. 5*G*,*H*). Together, our findings suggest that the activation of the Hh pathway is necessary for the differentiation of mESCs into certain types of LHA cells, including CART-negative MCH neurons and orexin neurons, and that such LHA cells are derived from ventral hypothalamic progenitors.

10.1523/ENEURO.0442-21.2022.f5-1 Extended Data Figure 5-1 Efficient induction of neural progenitors from mESCs in SFEBq culture with or without SAG. An SAG-treated (bottom) or untreated (top) mESC aggregate was immunostained for the general neural progenitor marker Sox1 on day 7. Nuclei were stained with DAPI. Scale bar: 100 μm. Download Figure 5-1, TIF file.

10.1523/ENEURO.0442-21.2022.f5-2 Extended Data Figure 5-2 Generation of MCH neurons in ES-Hypo in the presence of SAG. A, Representative images of an SAG-treated mESC aggregate immunostained for MCH and HuC/D on day 30. Scale bar: 20 μm. B, The percentage of MCH+HuC/D+ cells among total MCH+ or HuC/D+ cells. The values represent the mean ± SEM (n = 5 aggregates). Download Figure 5-2, TIF file.

10.1523/ENEURO.0442-21.2022.f5-3 Extended Data Figure 5-3 Co-expression of CART and NK3R in ES-Hypo in the presence of SAG. A, Representative images of an SAG-treated mESC aggregate immunostained for CART and NK3R on day 30. Scale bar: 20 μm. B, The percentage of CART+NK3R+ cells among total CART+ or NK3R^+^ cells. The values represent the mean ± SEM (*n *=* *4 aggregates). Download Figure 5-3, TIF file.

Since both CART^+^ and CART^−^ MCH neurons as well as orexin neurons were simultaneously produced in SAG-treated ES-Hypo, we reevaluated whether the MCH-orexin neuronal connectivity is specific to either subtype of MCH neurons. We performed triple immunostaining for MCH/orexin/CART but could not determine which subtypes of MCH neurons send fibers to orexin neurons, because CART-immunoreactivity was weak or undetectable in the fiber compartment (Fig. 5*E*). On the other hand, we observed both MCH^+^CART^+^ and MCH^+^CART^−^ cell bodies in contact with orexin-ir boutons (Fig. 5*I*). Thus, both subtypes of MCH neurons seem to receive orexinergic inputs in ES-Hypo.

### Progenitor origin of MCH neurons in ES-Hypo

Our results suggest that MCH neurons develop from multiple progenitor lineages which are specified through early dorsoventral patterning of the hypothalamus. To better understand the differentiation process of MCH neurons, we examined the expression patterns of regional markers as well as differentiation markers in ES-Hypo from days 7–22.

**Figure 6.**
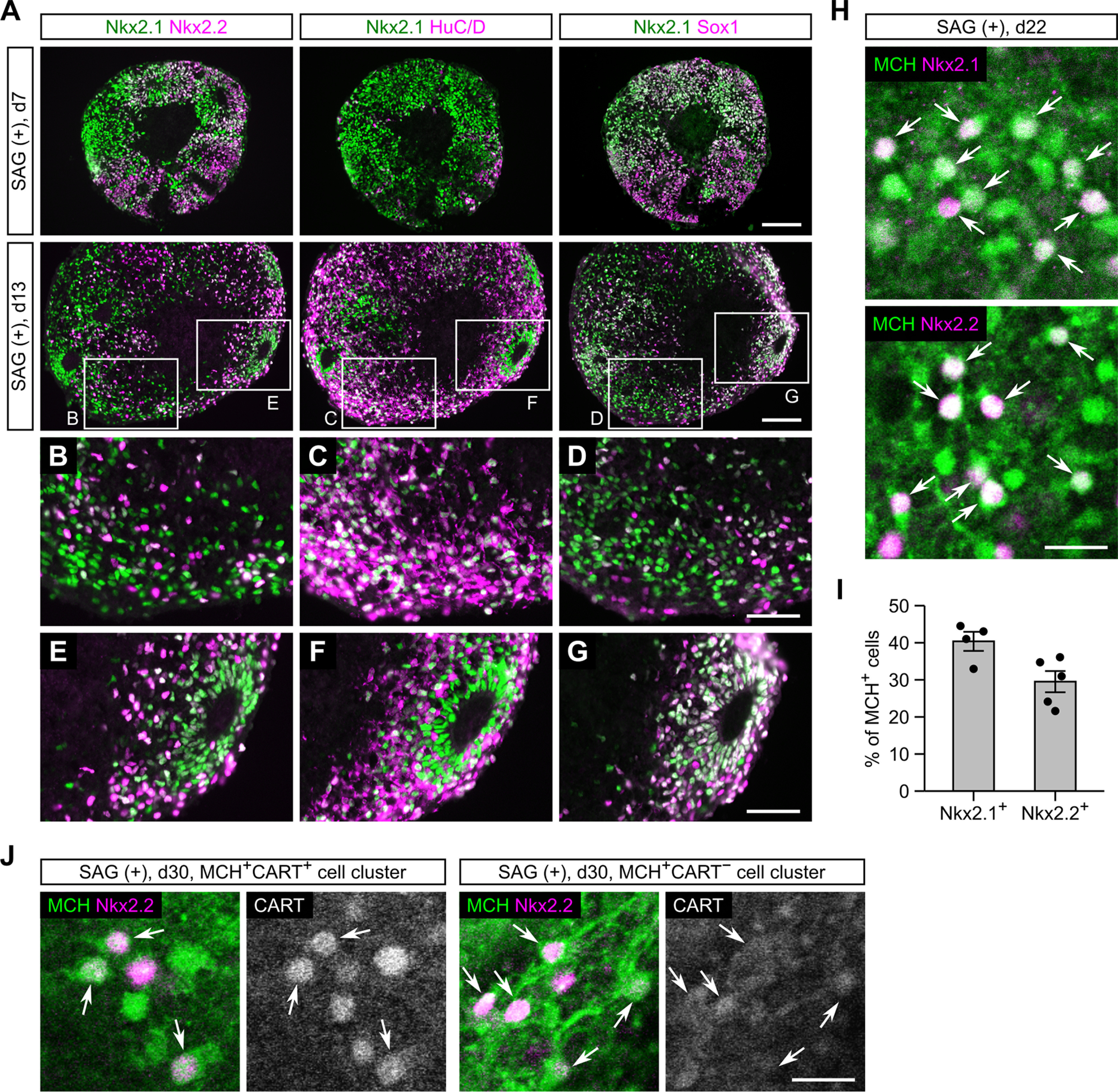
Characterization of neuronal differentiation in SAG-treated ES-Hypo. ***A–G***, Serial sections from SFEBq-cultured mESC aggregates (with SAG) on days 7 and 13. The sections were immunostained for Nkx2.1/Nkx2.2 (#1), Nkx2.1/HuC/D (#2), or Nkx2.1/Sox1 (#3). The day-13 aggregate contains a HuC/D^+^ neuron-dense area (***B–D***) and a Sox1^+^ rosette structure (***E–G***). Scale bars: 100 μm (***A***) and 50 μm (***B–G***). ***H***, Representative images of SAG-treated mESC aggregates immunostained for MCH/Nkx2.1 (top) and MCH/Nkx2.2 (bottom) on day 22. Arrows indicate double-positive cells. Scale bar: 20 μm. ***I***, The percentage of MCH-ir cells expressing Nkx2.1 or Nkx2.2 on day 22. *n *=* *4–5 aggregates per marker. ***J***, Triple immunostaing of SAG-treated mESC aggregates for MCH/CART/Nkx2.2 on day 30. Representative images of MCH^+^CART^+^ (left) and MCH^+^CART^−^ (right) cell clusters are shown, and MCH^+^Nkx2.2^+^ cells are indicated by arrows. Scale bar: 20 μm.

We first focused on SAG-treated ES-Hypo. On day 7, differentiating mESCs were mostly in the Nkx2.1^+^Sox1^+^ progenitor state and only partially positive for the neuronal marker HuC/D (Fig. 6*A*). On day 13, the robust expression of Nkx2.1 was still maintained, but there was a significant increase in HuC/D^+^ neurons, associated with downregulation of Sox1 (Fig. 6*A*,*C*,*D*). Most of the HuC/D^+^ neurons expressed Nkx2.1 (Fig. 6*C*), confirming that Nkx2.1^+^ progenitors are the primary source of postmitotic neurons in the presence of SAG. Indeed, we often observed Nkx2.1^+^Sox1^+^ rosette structures surrounded by HuC/D^+^ neurons (Fig. 6*F*,*G*), probably representing active sites of neurogenesis. The expression of Pax6 was completely absent on day 13 (data not shown), as on day 7 (Fig. 5*B*). The expression of Nkx2.2 was sustained from days 7–13 (Fig. 6*A*) and confined to roughly half of Nkx2.1^+^ cells, including HuC/D^+^ neurons (Fig. 6*B*) and Sox1^+^ rosette cells (Fig. 6*E*). Thus, two subtypes of Nkx2.1^+^ progenitors, Nkx2.1^+^Nkx2.2^+^ and Nkx2.1^+^Nkx2.2^−^, both undergo neurogenesis. On day 22, Nkx2.1 and Nkx2.2 were expressed in 40% and 30% of MCH-ir cells, respectively (Fig. 6*H*,*I*). We wondered whether the expression of Nkx2.2 is specific to either CART^+^ or CART^−^ MCH neurons, but Nkx2.2-immunoreactivity was clearly detected in both subtypes of MCH neurons (Fig. 6*J*). These results indicate that Nkx2.1^+^Nkx2.2^+^ progenitors directly generate both subtypes of MCH neurons in SAG-treated ES-Hypo.

**Figure 7.**
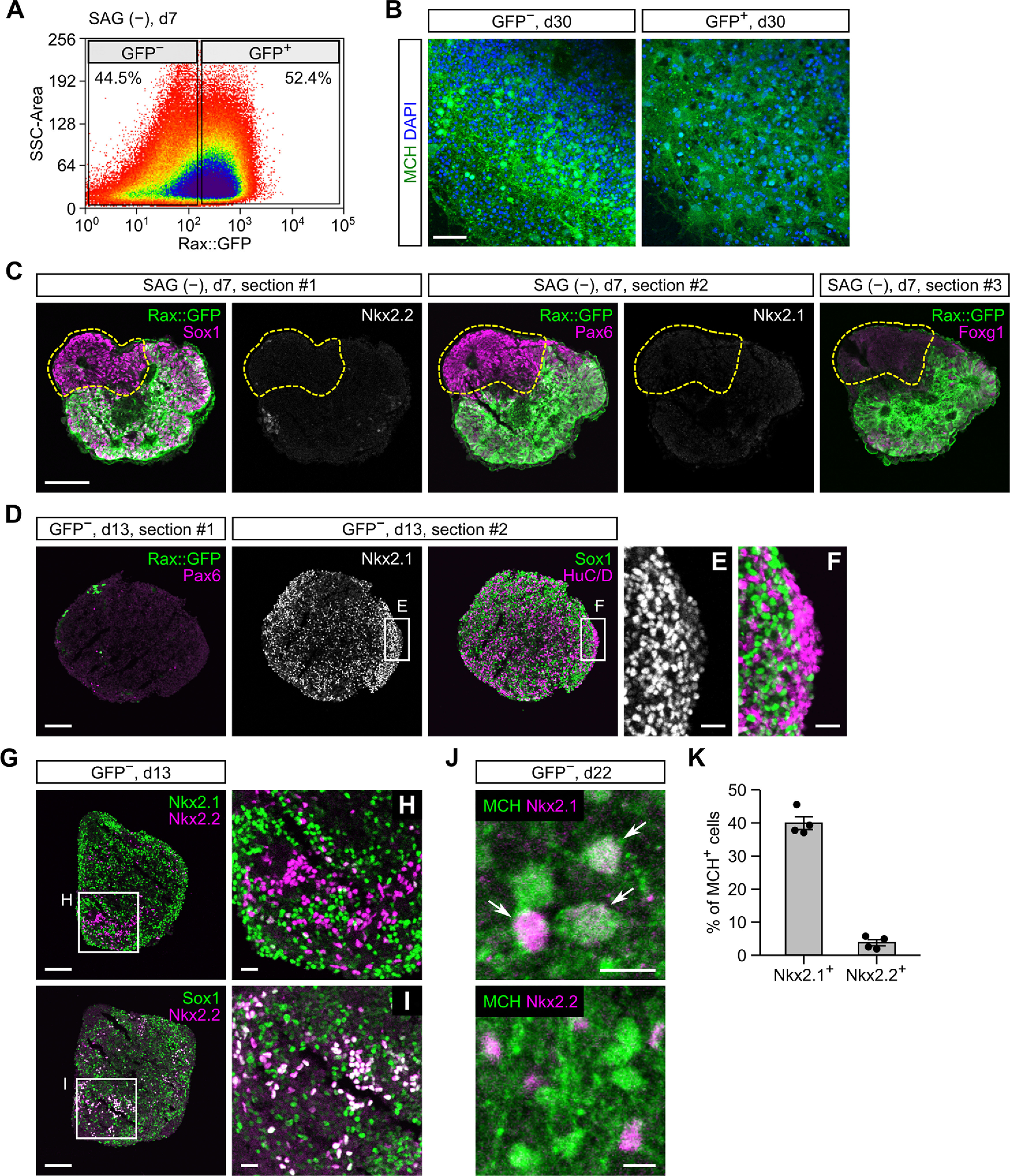
Characterization of neuronal differentiation in SAG-free ES-Hypo. ***A***, FACS sorting of Rax::GFP^+^ and GFP^−^ cells from SFEBq-cultured mESC aggregates (without SAG) on day 7. ***B***, Immunofluorescence images of Rax::GFP^+^ (right) and GFP^−^ (left) cell aggregates. FACS-sorted GFP^+^ and GFP^−^ cells were reaggregated and cultured until day 30 before staining for MCH. Nuclei were stained with DAPI. Scale bar: 50 μm. ***C***, Serial sections from a day-7 aggregate cultured without SAG. The sections were immunostained for GFP/Sox1/Nkx2.2 (#1), GFP/Pax6/Nkx2.1 (#2), or GFP/Foxg1 (#3). Scale bar: 100 μm. ***D–J***, Immunofluorescence analysis of GFP^−^ cell aggregates on days 13 and 22. Two serial sections from a day-13 aggregate were stained for GFP/Pax6 or Nkx2.1/Sox1/HuC/D (***D–F***). Two sections from a day-13 aggregate were stained for Nkx2.1/Nkx2.2 or Sox1/Nkx2.2 (***G–I***). Day-22 aggregates were stained for MCH/Nkx2.1 or MCH/Nkx2.2 (***J***, arrows indicate double-positive cells). Scale bars: 100 μm (***D***, ***G***), 20 μm (***E***, ***F***, ***H***, ***I***), and 10 μm (***J***). ***K***, The percentage of MCH-ir cells expressing Nkx2.1 or Nkx2.2 in GFP^−^ cell aggregates on day 22. *n *=* *4 aggregates per marker.

We next tested SAG-free ES-Hypo. During first week of culture, many mESCs differentiate into Rax^+^Pax6^+^ progenitors (Fig. 5*B*,*C*). However, it is difficult to trace the neuronal progeny of these progenitors because the expression of Rax/Pax6 was abolished in postmitotic cells in the hypothalamus (Wataya et al., 2008). We therefore performed FACS sorting of Rax::GFP^+^ and GFP^−^ cells on day 7 (Fig. 7*A*) to clarify whether Rax^+^Pax6^+^ progenitors are capable of producing MCH neurons. Unexpectedly, we found that the GFP^−^ but not GFP^+^ cell fraction robustly generated MCH-ir cells after neuronal differentiation (Fig. 7*B*). On day 7, Rax::GFP^−^ cells expressed Sox1/Pax6 but not Nkx2.1/Nkx2.2/Foxg1 (Fig. 7*C*). This expression profile represents a subpopulation of dorsal hypothalamic progenitors (Wataya et al., 2008) or prethalamic progenitors (Fig. 5*A*). We further analyzed the neuronal differentiation of the sorted Rax::GFP^−^ cells (i.e., Rax^−^Pax6^+^ progenitors). The expression of Pax6 was almost completely abolished in the GFP^−^ cell aggregates on day 13, but surprisingly, a great number of Nkx2.1^+^ cells were observed in the same samples (Fig. 7*D*). These Nkx2.1^+^ cells contained Sox1^+^ neural progenitors and HuC/D^+^ postmitotic neurons (Fig. 7*D–F*). Both populations were contiguous or intermingled, indicating that Nkx2.1^+^HuC/D^+^ neurons were directly produced by Nkx2.1^+^Sox1^+^ progenitors. Because Nkx2.1-immunoreactivity in HuC/D^+^ neurons was weaker than in neighboring Sox1^+^ progenitors (Fig. 7*E*,*F*), Nkx2.1 seems downregulated on neuronal differentiation. Nevertheless, more than half of HuC/D^+^ neurons were detected as Nkx2.1^+^, suggesting that Rax^−^Pax6^+^ early progenitors contribute to neurogenesis by supplying Nkx2.1^+^ intermediate progenitors rather than producing neurons by themselves. We also observed a small number of Nkx2.2^+^ cells, but most of them were Nkx2.1^−^/Sox1^+^ (Fig. 7*G–I*) and therefore probably represent a progenitor population distinct from the Nkx2.1^+^ cell lineage. When GFP^−^ cell aggregates were cultured until day 22, the expression of Nkx2.1 was observed in 40% of MCH-ir cells but that of Nkx2.2 was limited to ∼4% of MCH-ir cells (Fig. 7*J*,*K*). Taken together, our data suggest that MCH neurons can originate from Rax^−^Pax6^+^ early progenitors mainly through Nkx2.1^+^Nkx2.2^−^ intermediate progenitors in SAG-free ES-Hypo.

## Discussion

MCH neurons are preferentially located in the LHA, but they have no well-defined territory and are intermingled with other cell groups, such as orexin neurons (Hahn, 2010). This organization makes it hard to predict the embryonic progenitor domain as well as the developmental signals (e.g., morphogens and transcriptional factors) allocated for MCH neurons. ES-Hypo can partially resolve this difficulty; it is usable for screening culture conditions to induce specific hypothalamic lineages in a systematic, stepwise manner, and such trials should provide crucial information on the lineage specification and maturation. In the present study, we used this approach to gain insight into the mechanism of MCH neuron development. We found that ES-Hypo can produce several MCH-ir neurons that reproduce well-known features of native MCH neurons. Most importantly, we uncovered that the major neurochemical subpopulations of MCH neurons, the CART^+^ and CART^−^ MCH neurons, are differentiated from mESCs under the influence of Hh signaling.

The original report on ES-Hypo was primarily focused on the culture conditions to specify Rax^+^ hypothalamic progenitors and did not fully examine the neuronal fates of the progenitors (Wataya et al., 2008). The authors described that the Rax^+^ progenitors bearing a dorsal or ventral hypothalamic identity could generate vasopressin neurons or neurons observed in the ventromedial and arcuate nuclei of the hypothalamus, respectively. Subsequent reports have documented the occurrence of orexin neurons in ES-Hypo (Merkle et al., 2015) and its modified culture (Hayakawa et al., 2013); however, the present study provides the first characterization of MCH neurons in ES-Hypo. We found that a large fraction (37–81%) of neurons in ES-Hypo were immunopositive for MCH, regardless of whether ES-Hypo was derived from dorsal or ventral hypothalamic progenitors. This proportion seems to be much higher in comparison to previous reports on vasopressin neurons (6% of day-20 cells differentiated from Rax::GFP^+^ progenitors; Wataya et al., 2008). Indeed, we observed less copeptin-ir cells (copeptin is a peptide derived from the vasopressin precursor) in comparison to MCH-ir cells in ES-Hypo after four to five weeks of differentiation (data not shown). These data suggest that ES-Hypo generates a wide range of hypothalamic neurons, among which MCH neurons are a predominant population.

We examined the time course of neural progenitor specification and subsequent neurogenesis in ES-Hypo and showed that it follows the temporal pattern of fetal hypothalamic development. In addition to this overall similarity, MCH cells born in ES-Hypo reproduced morphologic changes observed *in vivo* for developing MCH neurons and acquired neurochemical phenotypes common to mature hypothalamic MCH neurons (the latter feature is discussed in detail below). Moreover, we found that orexin neurons developed later than MCH neurons in ES-Hypo and these cell groups appeared to form reciprocal connections. Both findings are reminiscent of native MCH and orexin neurons in the rodent hypothalamus (Bayer et al., 2002; Guan et al., 2002; Steininger et al., 2004; van den Pol et al., 2004; Díaz et al., 2014). Although it is unknown whether the MCH-orexin neuronal connections are specific to either of the subpopulations of MCH neurons *in vivo*, our results suggest that both CART^+^ and CART^−^ MCH neurons receive inputs from orexin neurons in ES-Hypo. A recent study succeeded in generating MCH and orexin neurons from mouse iPSCs using a culture protocol based on that for ES-Hypo (Seifinejad et al., 2019). The authors achieved neuronal differentiation within two weeks of culture by applying inductive factors, such as BMP7 and vitamin C, whereas we confirmed the generation of MCH and orexin neurons after longer culture of ES-Hypo without any inductive factors. Taken together, our data support the idea that ES-Hypo can recapitulate the developmental programs of MCH neurons and probably orexin neurons in an autonomous manner.

One of the most remarkable properties of native MCH neurons is their co-expression of multiple neurochemical molecules, including neuropeptides (CART and nesfatin-1) and markers for GABAergic and glutamatergic cells (GAD67 and VGLUT2). The expression of CART, nesfatin-1, and GAD67 in MCH neurons was previously detected at both mRNA and protein levels (Broberger, 1999; Vrang et al., 1999; Brailoiu et al., 2007; Sapin et al., 2010; Mickelsen et al., 2017; Noble et al., 2018). The expression of VGLUT2 was found in nearly all MCH neurons using VGLUT2-GFP reporter mice, single-cell transcriptomics, and ISH (Chee et al., 2015; Mickelsen et al., 2017, 2019; Schneeberger et al., 2018; Naganuma et al., 2019; Hung et al., 2020). It has been reported that VGLUT2 deletion in MCH neurons affects glucose metabolism, food reward, and REM sleep in mice (Schneeberger et al., 2018; Naganuma et al., 2019), suggesting functional glutamate signaling by MCH neurons. On the other hand, another study using VGLUT2-tdTomato mice (generated by mating VGLUT2-Cre mice to Ai14 Cre reporter mice) failed to detect tdTomato signals in MCH neurons (Blanco-Centurion et al., 2018). Our results based on double immunofluorescence staining indicated that mESC-derived MCH neurons largely co-express CART, nesfatin-1, GAD67, and VGLUT2. The co-expression of these markers has been confirmed in native MCH neurons at mRNA levels (Mickelsen et al., 2017); however, to our knowledge, this is the first report to demonstrate the simultaneous presence of endogenous GAD67 and VGLUT2 proteins in MCH neurons. Although the functional consequences of this neurochemical multiplicity remain to be elucidated, it has been shown that native MCH neurons can release GABA (Jego et al., 2013) and glutamate (Chee et al., 2015), as well as MCH (Noble et al., 2018). Thus, the present study not only supports the neurochemical similarity between mESC-derived and native MCH neurons, but also provides additional evidence of the dual GABAergic-glutamatergic phenotype of MCH neurons.

**Figure 8.**
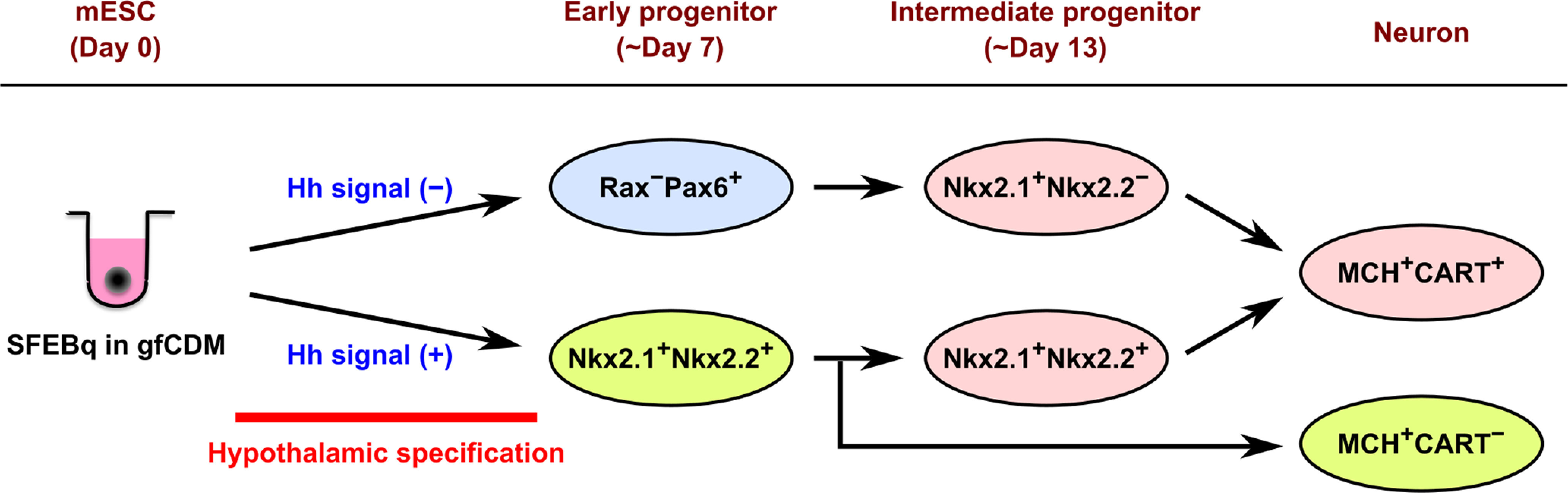
Schematic diagram of the different progenitor origins of MCH neurons suggested in the current study. SFEBq culture of mESCs in gfCDM with or without exogenous Hh signals can generate Nkx2.1^+^Nkx2.2^+^ ventral hypothalamic progenitors or Rax^−^Pax6^+^ dorsal hypothalamic/prethalamic progenitors, respectively, within a week. The Nkx2.1^+^Nkx2.2^+^ early progenitors directly produce MCH^+^CART^−^ neurons and Nkx2.1^+^Nkx2.2^+^ intermediate progenitors, the latter of which generate MCH^+^CART^+^ neurons. The Rax^−^Pax6^+^ early progenitors also differentiate into MCH^+^CART^+^ neurons through Nkx2.1^+^Nkx2.2^−^ intermediate progenitors.

The most striking finding of our study is that CART-negative MCH neurons substantially developed in ES-Hypo only when the Hh agonist SAG was added to the differentiation media. In the embryonic mouse brain, most MCH^+^CART^−^ neurons are born at the initial phase of neurogenesis (E9–E10) and followed by the generation of MCH^+^CART^+^ neurons (E11–E12; Croizier et al., 2010). This birth order is reflected in the final localization of those neurons in the adult brain. Earlier born MCH^+^CART^−^ neurons are predominantly located in the dorsal-lateral part of the caudal LHA close to the cerebellar peduncle, although a minor fraction of them are found in the perifornical region of the LHA at more caudal levels (Croizier et al., 2010). Later born MCH^+^CART^+^ neurons are mainly distributed in the rostral and medial hypothalamic portions, but also in additional locations, including the rostral zona incerta, the dorsal-lateral part of the caudal LHA, and the ventral capsule of the posterior hypothalamic nucleus (Croizier et al., 2010). It remains unknown whether these subtypes of MCH neurons originate from a common or distinct progenitors. On the other hand, a previous study has shown that early-born MCH^+^ cells in the E13–E15 rat brain appear to come from the Nkx2.1/Nkx2.2 co-expressing region in the caudal hypothalamic neuroepithelium (Croizier et al., 2011; Fig. 5*A*). The corresponding region is also proposed as an origin of early MCH cells in the fetal mouse brain (Díaz et al., 2014). Consistent with these *in vivo* data, SFEBq-cultured mESCs differentiated into Nkx2.1^+^Nkx2.2^+^ hypothalamic progenitors and then generated MCH neurons, including both CART^+^ and CART^−^ cells, in the presence of SAG. Under this condition, most mESCs were specified into Nkx2.1^+^ ventral hypothalamic progenitors by Hh signaling, like the patterning event in the hypothalamic anlage (Xie and Dorsky, 2017). Only half of the Nkx2.1^+^ progenitors co-expressed Nkx2.2, but we observed co-localization of Nkx2.2 with both subtypes of MCH neurons after terminal differentiation. Based on this result, we concluded that Nkx2.1^+^Nkx2.2^+^ progenitors are an origin of both CART^+^ and CART^−^ MCH neurons in SAG-treated ES-Hypo. We cannot rule out the possibility that other types of neural progenitors also contribute to the production of MCH neurons, as the expression of Nkx2.1/Nkx2.2 was limited to 30–40% of MCH neurons (Fig. 6*I*). However, the expression of the Nkx genes seems downregulated on terminal differentiation of MCH neurons (Croizier et al., 2011), suggesting that most MCH neurons are produced by Nkx2.1^+^Nkx2.2^+^ progenitors in the presence of SAG. It is currently unclear how Nkx2.1^+^Nkx2.2^+^ progenitors generate the two subtypes of MCH neurons. A plausible hypothesis is that CART^−^ and CART^+^ MCH neurons are derived from early and intermediate stages of Nkx2.1^+^Nkx2.2^+^ progenitors, respectively, according to the birth order for *in vivo* MCH neurons (Fig. 8). This idea is consistent with our observation that both subpopulations of MCH neurons were generally found in discrete clusters and not intermingled with each other in ES-Hypo (Fig. 5*E*). In this case, a stage-specific phenotype of Nkx2.1^+^Nkx2.2^+^ progenitors may determine the phenotype of their descendant MCH neurons. Alternatively, extracellular factors may influence late generated MCH neurons to express CART, as suggested previously (Cvetkovic et al., 2004).

In the present study, we only performed SAG treatment in a continuous manner (from day 4 onward) and therefore could not clarify whether Hh signaling has an effect not only on ventralizing hypothalamic progenitors but also on their subsequent terminal differentiation. In this respect, a genetic study has revealed that the elimination of the functional Shh expression in the developing diencephalon severely impairs the production of MCH neurons, especially in the LHA (Szabó et al., 2009), wherein MCH^+^CART^−^ neurons are predominantly localized. It has also been reported that the MCH mRNA level was decreased by 90% in E13 mouse embryos after pregnant mice were injected with an Hh inhibitor on E11 (Croizier et al., 2011), when most presumptive MCH^+^CART^−^ neurons have already been born in embryos (Croizier et al., 2010). These previous findings provide evidence that Hh signaling is required for terminal differentiation and/or survival of MCH^+^CART^−^ neurons.

Without SAG treatment, SFEBq-cultured mESCs differentiated into Rax^+^Pax6^+^ dorsal hypothalamic progenitors or Rax^−^Pax6^+^ dorsal hypothalamic/prethalamic progenitors within the first week of culture. Under this condition, terminally differentiated MCH neurons were almost completely CART^+^. Through FACS sorting and subsequent differentiation analysis, we found that Rax^−^Pax6^+^ progenitors are an early origin of MCH^+^CART^+^ neurons in SAG-free ES-Hypo. These early progenitors next generate the second form of neural progenitors (Nkx2.1^+^Nkx2.2^−^), and this group of intermediate progenitors directly produces MCH neurons (Fig. 8). Although the regional identity of these intermediate progenitors remains unclear, a possible candidate is the perimamillary/periretromamillary (PM/PRM) region in the prosomeric hypothalamic model (Díaz et al., 2014). This region was identified as the second site of occurrence of MCH^+^ cells, more ventral to the first site (i.e., the Nkx2.1/Nkx2.2-positive region), by using the Allen Developmental Mouse Brain Atlas (Díaz et al., 2014). The PRM/PM area expresses Nkx2.1 but not Nkx2.2 (Díaz et al., 2014), consistent with the phenotype of the intermediate progenitors identified in SAG-free ES-Hypo.

In summary, we propose three different progenitor origins of MCH neurons in ES-Hypo (Fig. 8): (1) Nkx2.1^+^Nkx2.2^+^ early progenitors, (2) Nkx2.1^+^Nkx2.2^+^ intermediate progenitors, and (3) Nkx2.1^+^Nkx2.2^−^ intermediate progenitors. The first group selectively produces CART-negative MCH neurons, and the others CART-positive MCH neurons. The multiple progenitor origins of MCH neurons can be supported by recent transcriptome studies. Kim et al. (2020) performed clustering of embryonic mouse LHA cells (at E11–E13) using single-cell RNA-sequencing and identified 13 subclusters of young postmitotic neurons. Among them, five subclusters expressed both MCH and CART mRNAs and one subcluster MCH mRNA alone, implying that MCH neurons develop through multiple pathways. A similar transcriptional diversity was also found in hypothalamic POMC precursors (immature POMC^+^ neurons), and it was shown that distinct subclusters of POMC precursors finally develop into different phenotypes of arcuate neurons (Yu et al., 2022). In light of these findings, it is plausible that CART^+^ and CART^−^ MCH neurons are developed from distinct progenitors. Our results also suggested that CART-positive MCH neurons originate from two distinct progenitor lineages, which are specified in the presence or absence of Hh signaling (Fig. 8). This may account for the relatively wide distribution of MCH^+^CART^+^ neurons compared with MCH^+^CART^−^ neurons in the adult brain. Further studies, using a lineage tracing approach are needed to better understand the ontogeny of MCH neurons.

Another notable effect of SAG on ES-Hypo was the increased production of orexin neurons. Orexin-ir cells were observed only occasionally in the absence of SAG but were more reproducibly observed in the SAG-treated samples. Although the ontogeny of orexin neurons remains largely unknown, the LIM homeobox transcription factor Lhx9 has been identified as a critical inducer of the orexin neuron specification in zebrafish and developing mice (Dalal et al., 2013; Liu et al., 2015). In the latter species, the expression of Lhx9 occurs in the mantle layer of the tuberal hypothalamus during the neurogenic period and then colocalizes with a subset of orexin neurons in the LHA (Rétaux et al., 1999; Shimogori et al., 2010; Sokolowski et al., 2015). It has also been revealed that Shh signaling is indispensable for the development of orexin neurons as well as Lhx9^+^ cells in the mouse hypothalamus (Szabó et al., 2009; Shimogori et al., 2010). These previous data and our current findings strongly suggest that orexin neurons primarily arise from ventral hypothalamic progenitors under the control of Hh signaling. In this view, the stochastic induction of orexin cells without SAG is attributable to a small number of Nkx2.1^+^ progenitors (Fig. 5*B*,*C*). In comparison to the abundant production of MCH neurons in ES-Hypo, the number of co-existing orexin neurons was much smaller, even in the presence of SAG (<15 cells on day 30). In rodents, most MCH neurons appear before birth; however, the number of orexin neurons as well as orexin immunoreactivity increase in the postnatal period (Yamamoto et al., 2000; Steininger et al., 2004; Ogawa et al., 2017). These observations suggest that the generation of orexin neurons in ES-Hypo may be promoted by extending the culture period in addition to SAG treatment. However, with more than five weeks of mESC differentiation, there was a massive expansion of residual undifferentiated cells, which negatively affected the survival of differentiated neurons (data not shown).

In conclusion, ES-Hypo can recapitulate the developmental process of MCH neurons *in vitro* and therefore be a powerful tool to dissect the molecular mechanisms underlying this process. We also demonstrated that the Hh signaling pathway is a key organizer of the CART^+^ and CART^−^ subpopulations of MCH neurons. These neurochemical subpopulations have also been found in the human hypothalamus (Menyhért et al., 2007). Hence, future comparative studies on MCH neurons derived from rodent and human stem cell lines may help clarify whether Hh signaling plays a conserved role in establishing the heterogeneity of MCH neurons in mammals.

## Synthesis

Reviewing Editor: Lynda Erskine, University of Aberdeen Decisions are customarily a result of the Reviewing Editor and the peer reviewers coming together and discussing their recommendations until a consensus is reached. When revisions are invited, a fact-based synthesis statement explaining their decision and outlining what is needed to prepare a revision will be listed below. The following reviewer(s) agreed to reveal their identity: Sophie Croizier.

Your manuscript has been reviewed by two experts in the field and considered potentially suitable for publication in eNeuro. However, while the manuscript potentially provides new insights into the developmental pathways leading to the differentiation of MCH neurons in the lateral hypothalamic area, there are a number of concerns raised by the reviewers that need to be addressed. This will involve both substantial changes to the text as well as new experiments. Below is a synthesis of the reviews detailing all of the points that we are agreed need to be addressed in a revised manuscript:

Major points:

1. What are the pros and cons of using SFEBq culture system, as compared with the other in vitro culture system? It would be better to perform benchmark test to compare different culture systems in Figure 1. Please refer to the following papers:

• Wang et al., Differentiation of hypothalamic-like neurons from human pluripotent stem cells.

• Rajamani et al., Super-obese patient-derived iPSC hypothalamic neurons exhibit obesogenic signatures and hormone response.

2. The evidence to indicate the different origins of MCH+CART+ to MCH+CART- neurons is not convincing. There is neither compelling evidence to show that activation of Hh pathway could distinguish ventral from dorsal hypothalamic progenitor specification in the previous studies. The authors should use specific markers to label dorsal and ventral hypothalamic progenitors.

The authors should also comment more on the origin of CART+ and CART- MCH neurons and their final localization in the LHA, medial versus lateral, respectively in mice.

3. What are the morphological, histological and neurochemical differences between MCH+CART+ to MCH+CART- neurons?

4. The authors stated that the induced MCH neurons recapitulated the morphological, histological, and neurochemical phenotypes of native MCH neurons in vivo. It would be better to systematically compare the molecular, morphology and electrophysiological properties of MCH neurons that are generated in vivo and in vitro.

4. Deeper analysis of the expression of Pax6/Nkx2.1 should be performed over time (between d7 and d22 when MCH appears). Related to this the authors should discuss the fact that MCH neurons have been shown to be generated in a Pax6 negative and Nkx2.1/Nkx2.2 positive region in vivo in rats, and here they are generated in a Rax+Pax6+ area.

5. The authors should assess the NK3 receptor expression in addition of CART as in vivo this sub-population co-express both.

6. The author should assess whether the interconnections of orexin/MCH neurons was specific of CART+ or CART- MCH neurons.

Additional points:

1. The 2 extended data are very informative, but the sagittal view of the embryo and the orientation of the brain should be added to one of the main figures. It is sometimes very confusing as the embryonic axis used in this study is based on adult brain. and with the cephalic flexure of the embryos, it might be hard to follow for most of the readers.

For more clarity, please refer as often as necessary to the figure illustrating the orientation of the embryo axis

2. For most of the graphs, statistical analysis have been performed properly. However, a statistical analysis for the graphs shown in Figures 1G, 2A, and 3B should be performed.

3. In the methods, please specify if diffuse EdU staining was also obtained, and, if yes, were they quantified/considered as EdU-positive.

4. Please explain what KSR is and what it is supposed to change in the culture.

5. A better description should be provided of what the authors have called long and small diameters. As written it is confusing as they are evaluated at both d22 and d36 where we can find either smaller or bigger neurons. Are these diameters evaluated on the same neuron? One represents the ‘length’ and the other the ‘width’, as the neurons are angular-shaped? A schematic would be very useful.

6. Line 238, ’increased from day 9’. Can this be stated with certainty as birth at E8 has not been evaluated. Indeed, it is maybe already the case at E8.

7. Line 280 - The meaning of “essentially immunonegative” needs to be made clearer. A few double-positive MCH/Orexin were observed. What about the frequency?

8. The single cell analysis performed by Michelsen et al. eNeuro 2017 should be cited (in the discussion, lines 404, 405, and in the results part paragraph line 286)

9. The data can be discussed in light of the single cells analysis made by Malcom Low available in BioRXiv on POMC neurons and their trajectories.

10. Typo: legend figure 1, the statistics describe **** while it is *** in figure 1D

## Author Response

Dear Dr. Erskine,

Thank you very much for your e-mail and review of the manuscript (eN-NWR-0442-21) that we sent on October 18, 2021. We thank two reviewers for providing constructive comments regarding the improvement of the original manuscript. We have carried out additional experiments to respond the reviewers’ major concerns. Newly generated data provide evidence to indicate the multiple origins of MCH neurons.

Please find the uploaded revised manuscript. All changes have been made in response to the reviewers’ suggestion. Our itemized response to the reviewers’ comments is listed below.

Major points:

Q1. What are the pros and cons of using SFEBq culture system, as compared with the other in vitro culture system? It would be better to perform benchmark test to compare different culture systems in Figure 1. Please refer to the following papers:

• Wang et al., Differentiation of hypothalamic-like neurons from human pluripotent stem cells.

• Rajamani et al., Super-obese patient-derived iPSC hypothalamic neurons exhibit obesogenic signatures and hormone response.

A1. We appreciate valuable suggestion of the reviewer. As pointed out, 2D culture systems distinct from SFEBq have been reported for hESCs/hiPSCs by Wang et al. and Rajamani et al. We modified the Introduction and cited these papers (P.4, lines 70 - 75). However, we aimed to analyze the development of MCH neurons using mESCs, as described in the Introduction. In most cases, the differentiation protocols for hESCs/iPSCs and mESCs are not interchangeable. For instance, the original SFEBq method is not directly applicable to hESCs/hiPSCs and significant modifications are required (Ogawa et al., 2018). Currently, SFEBq is the only established method for hypothalamic differentiation of mESCs and therefore we used this method. This description was added in the Introduction (P.5, lines 81 - 83).

Q2. The evidence to indicate the different origins of MCH+CART+ to MCH+CART- neurons is not convincing. There is neither compelling evidence to show that activation of Hh pathway could distinguish ventral from dorsal hypothalamic progenitor specification in the previous studies. The authors should use specific markers to label dorsal and ventral hypothalamic progenitors.

The authors should also comment more on the origin of CART+ and CART- MCH neurons and their final localization in the LHA, medial versus lateral, respectively in mice.

A2. In the original paper of SEFBq (Wataya te al., 2008), it has been shown that dorsal hypothalamic progenitors (Rax+Pax6+) are induced without Hh agonists, whereas ventral hypothalamic progenitors (Rax+Nkx2.1+) are induced by adding Shh. This is described in the Results (P.18, lines 372 - 374). We reproduced this result using the Hh agonist SAG, instead of Shh (Fig. 5B, C). We also found another type of ventral hypothalamic progenitors (Nkx2.1+Nkx2.2+) in the presence of SAG (Fig. 5D). To clearly show the regional specificity of above markers, the sagittal view of the embryo (modified from the original version of Extended Data Fig. 5-1) was inserted in Figure 5A. We also carried out immunostaining for Sox1 (a general neural progenitor marker) to demonstrate that SFEBq culturing selectively induce neural progenitors with or without SAG (P.19, lines 381 - 383; Extended Data Fig. 5-1).

In the Discussion (P.27 - 28, lines 543 - 578; P.29 - 30, lines 590 - 621), we commented on the birth order, localization, and origin of CART+ and CART- MCH neurons. In this part, we provided a detailed discussion on the multiple origins of MCH neurons based on the data from additional experiments (please see answer #5).

Q3. What are the morphological, histological and neurochemical differences between MCH+CART+ to MCH+CART- neurons?

A3. As explained in the next answer, we did not aim to perform a detailed characterization of phenotypic differences between MCH+CART+ to MCH+CART- neurons. However, we examined the expression of NK3 receptor (NK3R) because it is another well-known marker for the MCH+CART+ subtype. We found almost complete overlap of CART and NK3R in SAG-treated ES-Hypo, indicating that NK3R is expressed in MCH+CART+ but not MCH+CART- neurons (P.19 - 20, lines 399 - 405; Extended Data Fig. 5-3).

Q4. The authors stated that the induced MCH neurons recapitulated the morphological, histological, and neurochemical phenotypes of native MCH neurons in vivo. It would be better to systematically compare the molecular, morphology and electrophysiological properties of MCH neurons that are generated in vivo and in vitro.

A4. As suggested by the reviewer, a systematic comparison between in vivo and in vitro neurons would be important for investigating the phenotypic maturation of induced MCH neurons. However, many additional experiments are required for such comparison because few studies have defined the phenotypic differences between MCH neuron subtypes (i.e., CART+ or CART-) in vivo. In the current study, we primarily focused on analyzing the differentiation/specification process of the MCH neuron subtypes rather than their maturation process. We therefore did not make an extensive comparison of the neuronal phenotypes between in vivo and in vitro, but only tested whether general and well-known features of native MCH neurons are reproduced by induced MCH neurons. This analysis includes evaluations of the (1) morphological maturation, (2) neuronal connectivity, and (3) neuropeptide/neurotransmitter profile, as follows.

(1) Morphological maturation. Developing MCH neurons in vivo exhibit an enlargement of cell size and a shift of cell shape from round to angular one. These morphological changes have already been confirmed in our original manuscript (Fig. 2). Another hallmark of morphological maturation in vivo is an increase in neurite number, which results in a shift from unipolar/bipolar to multipolar morphology. Based on this, we additionally examined neurite number in induced MCH neurons in the dissociation culture and found that multipolar neurons increase over time (P.15 - 16, lines 305 - 312; Fig. 2D, E).

(2) Neuronal connectivity. Many studies have provided evidence of reciprocal connections between MCH and orexin neurons in the LHA, and we have shown putative connections between induced MCH and orexin neurons in the original manuscript (Fig. 3).

(3) Neuropeptide/neurotransmitter phenotype. We have shown that induced MCH neurons express a set of neuropeptide/neurotransmitter markers known to be expressed in native neurons (Fig. 4).

Based on the reviewer’s comment, we thought that our statement “the morphological, histological, and neurochemical phenotypes” is ambiguous. We therefore rephrased it as “the morphological maturation, neuronal connectivity, and neuropeptide/neurotransmitter phenotype” in the revised manuscript (P.1, lines 7 - 8; P.5, lines 87 - 88).

We did not perform electrophysiological experiments as suggested by the reviewer but evaluated the functionality of induced MCH neurons through calcium imaging. This additional experiment showed that induced MCH neurons are activated by KCl and glutamate but not by GABA, confirming that they are functional neurons (P.15 - 16, lines 313 - 315; Extended Data Fig. 2-1). The method of calcium imaging was also described in the revised manuscript (P.9 - 10, lines 173 - 195).

Q5. Deeper analysis of the expression of Pax6/Nkx2.1 should be performed over time (between d7 and d22 when MCH appears). Related to this the authors should discuss the fact that MCH neurons have been shown to be generated in a Pax6 negative and Nkx2.1/Nkx2.2 positive region in vivo in rats, and here they are generated in a Rax+Pax6+ area.

A5. We appreciate helpful suggestion of the reviewer. As suggested, we analyzed the expression of Pax6/Nkx2.1 on days 7, 13, and 22. We additionally tested the expression of Nkx2.2 and general differentiation markers (Sox1 and HuC/D).

In the presence of SAG (Fig. 6), the expression of Pax6 was completely absent, but that of Nkx2.1 and Nkx2.2 was maintained in postmitotic neurons on day 13 and in MCH neurons on day 22. Although Nkx2.2 was expressed in about half of Nkx2.1+ cells, both MCH+CART+ and MCH+CART- cells expressed Nkx2.2. We therefore concluded that Nkx2.1+Nkx2.2+ progenitors are an origin of MCH neurons.

In the absence of SAG (Fig. 7), we performed FACS sorting of Rax::GFP+ and GFP- cells on day 7 to trace the neuronal fate of Rax+Pax6+ progenitors, because the expression of Rax and Pax6 are abolished in postmitotic neurons. Unexpectedly, we found that sorted GFP- cells selectively generate MCH neurons. These GFP- cells exhibited a marker profile of dorsal hypothalamic progenitors or prethalamic progenitors (Pax6+/Rax-/Nkx2.1-/Nkx2.2-/Foxg1-). We then examined the expression of Pax6/Nkx2.1 and other markers during the differentiation of sorted GFP- cells and found that Pax6+Rax- early progenitors generate Nkx2.1+Nkx2.2- intermediate progenitors, which directly produce MCH neurons.

These data were presented in the last part of the Results (P.20 - 22, lines 421 - 468). Based on the results, we provided a deeper discussion on the origin of MCH neurons (P.27 - 28, lines 543 - 578; P.29 - 30, lines 590 - 621) and proposed three different progenitor origins of MCH neurons (Fig. 8). Because of these important findings, Abstract was also modified (P.1, lines 11 - 14).

The method of FACS sorting was described along with that of flow cytometry (P.8, lines 147 - 159). Since we cultured FACS-sorted cells, the cell culture part of the method section was also modified (P.6 - 7, lines 115 - 117, 122 - 127).

Q6. The authors should assess the NK3 receptor expression in addition of CART as in vivo this sub-population co-express both.

A6. We assessed NK3R expression, as mentioned in answer #3.

Q7. The author should assess whether the interconnections of orexin/MCH neurons was specific of CART+ or CART- MCH neurons.

A7. To address the issue pointed out by the reviewer, we performed triple immunostaining for MCH/orexin/CART in SAG-treated samples. We could not determine which subtypes of MCH neurons project to orexin neurons because CART-immunoreactivity was weak or undetectable in the fiber compartment. On the other hand, we identified both MCH+CART+ and MCH+CART− cell bodies in contact with orexin-ir boutons, suggesting that both subtypes of MCH neurons can receive inputs from orexin neurons. These data were inserted in the Results (P.20, lines 412 - 419; Fig. 5I). We also commented on this finding in the discussion (P.25, lines 508 - 510).

Additional points:

Q1. The 2 extended data are very informative, but the sagittal view of the embryo and the orientation of the brain should be added to one of the main figures. It is sometimes very confusing as the embryonic axis used in this study is based on adult brain. and with the cephalic flexure of the embryos, it might be hard to follow for most of the readers.

For more clarity, please refer as often as necessary to the figure illustrating the orientation of the embryo axis.

A1. As mentioned in the above, the sagittal view of the embryo (modified from the original version of Extended Data Fig. 5-1) was inserted in Figure 5A and referred as necessary.

Q2. For most of the graphs, statistical analysis have been performed properly. However, a statistical analysis for the graphs shown in Figures 1G, 2A, and 3B should be performed.

A2, We performed multiple comparison using Dunnett test in Figures 1G and 2A. However, we noticed that data presented in Figure 3B contains missing values in some data points. We therefore replaced the original graph with a two-bar graph that simply compares gene expression between days 14 and 31. We did not aim to determine the detailed time course of orexin gene expression because the timing of orexin expression was shown by immunohistochemistry. Thus, qRT-PCR data in Figure 3B was presented to demonstrate the increased gene expression on day 31 (P.17, lines 334 - 336). Welch’s t test was used for statistical analysis in Figure 3G. For qRT-PCR data in Figures 2A and 3B, log10-transformed data were used for statistical analysis to reduce variability of data. Because of this, qRT-PCR data were plotted in log10 scale. The results of the above statistical analyses were added in Table 2. We also modified sentences in the method of statistical analysis (P.13, lines 257 - 260).

Q3. In the methods, please specify if diffuse EdU staining was also obtained, and, if yes, were they quantified/considered as EdU-positive.

A3. We observed a part of HuC/D+ cells exhibiting diffuse EdU staining, and they were also considered as EdU+ cells. This is described in the methods (P.12, lines 240 - 241).

Q4. Please explain what KSR is and what it is supposed to change in the culture.

A4. As indicated, we explained about KSR and its action in the Results (P.14, lines 278 - 284).

Q5. A better description should be provided of what the authors have called long and small diameters. As written it is confusing as they are evaluated at both d22 and d36 where we can find either smaller or bigger neurons. Are these diameters evaluated on the same neuron? One represents the ’length’ and the other the ’width’, as the neurons are angular-shaped? A schematic would be very useful.

A5. We appreciate helpful suggestion of the reviewer. Long and short diameters were evaluated on the same neuron. We explained about the definition of these diameters using a representative image of MCH-ir cells (Fig. 2C).

Q6. Line 238, ’increased from day 9’. Can this be stated with certainty as birth at E8 has not been evaluated. Indeed, it is maybe already the case at E8.

A6. As per the reviewer’s suggestion, we modified the sentences in the Results (P.15, lines 286 - 289).

Q7. Line 280 - The meaning of “essentially immunonegative” needs to be made clearer. A few double-positive MCH/Orexin were observed. What about the frequency?

A7. As indicated, we described the frequency of double-positive cells in the Results (< 10%; P.17, lines 337 - 340).

Q8. The single cell analysis performed by Michelsen et al. eNeuro 2017 should be cited (in the discussion, lines 404, 405, and in the results part paragraph line 286).

A8. As per the reviewer’s suggestion, we cited the paper by Mickelsen et al. eNeuro 2017 in the Results (P.17, line 349) and Discussion (P.26, lines 532 - 533).

Q9. The data can be discussed in light of the single cells analysis made by Malcom Low available in BioRXiv on POMC neurons and their trajectories.

A9. As per the reviewer’s suggestion, we referred to the paper by Malcom Low (Yu et al., Elife, 2022) in the discussion on the origin of MCH neurons (P.30, lines 612 - 615).

Q10. Typo: legend figure 1, the statistics describe **** while it is *** in figure 1D

A10. As indicated, we corrected *** to **** in Figure 1D.
