# Supplementary figures and images for: Characterization of Hypothalamic MCH Neuron Development in a 3D Differentiation System of Mouse Embryonic Stem Cells
Source: eNeuro. 2022 Apr 26;9(2):ENEURO.0442-21.2022. doi: 10.1523/ENEURO.0442-21.2022 (PMC9047030; doi:10.1523/ENEURO.0442-21.2022)

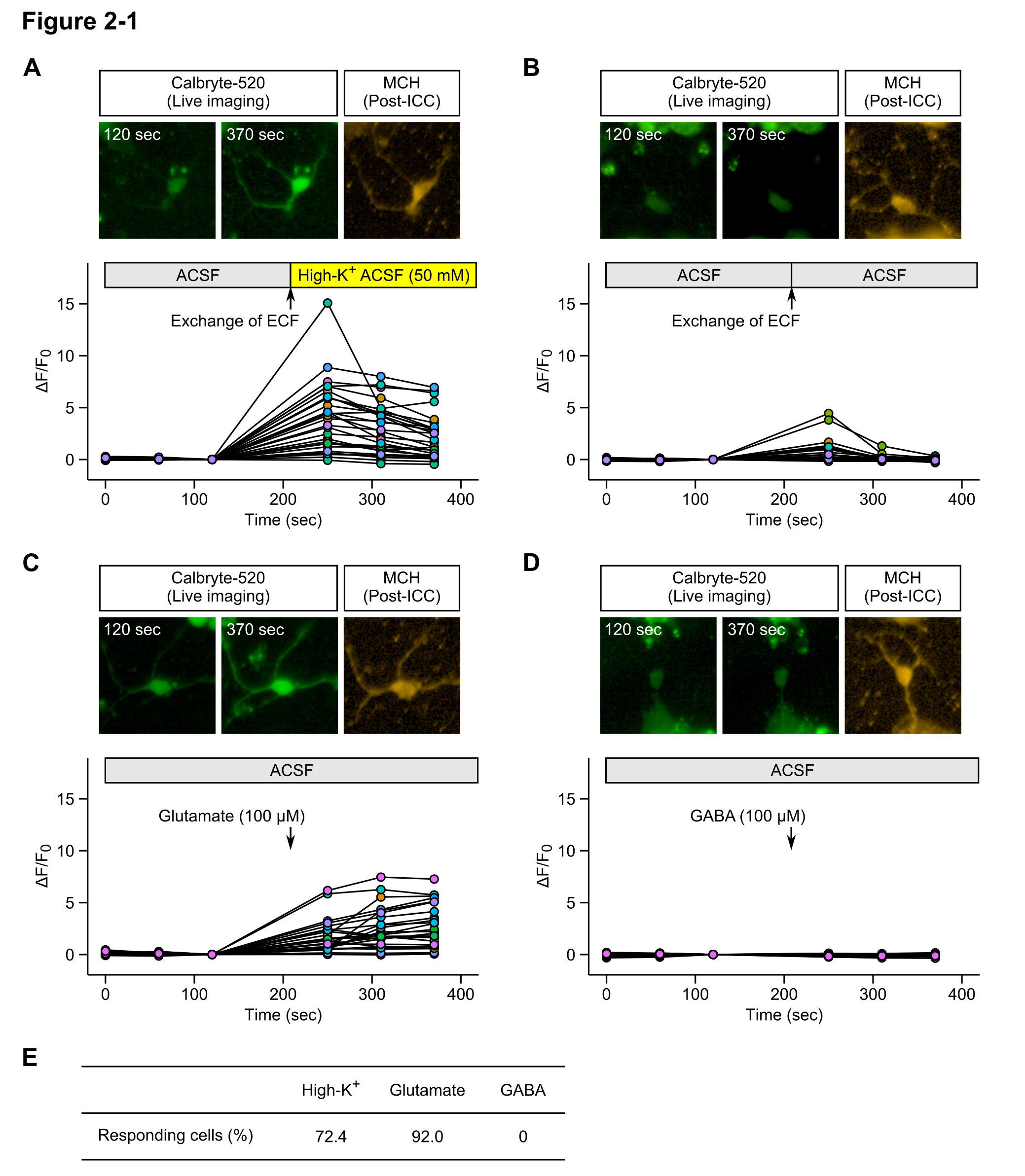

Supplement: Extended Data Figure 2-1 — Calcium imaging of mESC-derived MCH cells in the dissociation culture on day 36. A, Ca2+ response to high-K+ stimulation. The graph shows the time course of fluorescence signals from respective MCH cells (n = 29), which were loaded with Calbryte-520. ECF was changed from ACSF to 50 mm KCl-containing ACSF as indicated by arrow. Representative images of an MCH cell are shown on top of the graph; Calbryte-520 fluorescence at 120 s (left) and 370 s (middle), and postimmunocytochemistry (ICC) for MCH (right). B, A control experiment for high-K+ stimulation (n = 29). ECF were changed from ACSF to ACSF. C, D, Ca2+ response to glutamate (C, n = 25) or GABA (D, n = 36). The reagents were applied at final concentration of 100 μm as indicated by arrows. E, The percentage of MCH+ cells responding to high-K+, glutamate, or GABA. Download Figure 2-1, TIF file. [file enu-eN-NWR-0442-21-s01.tif]

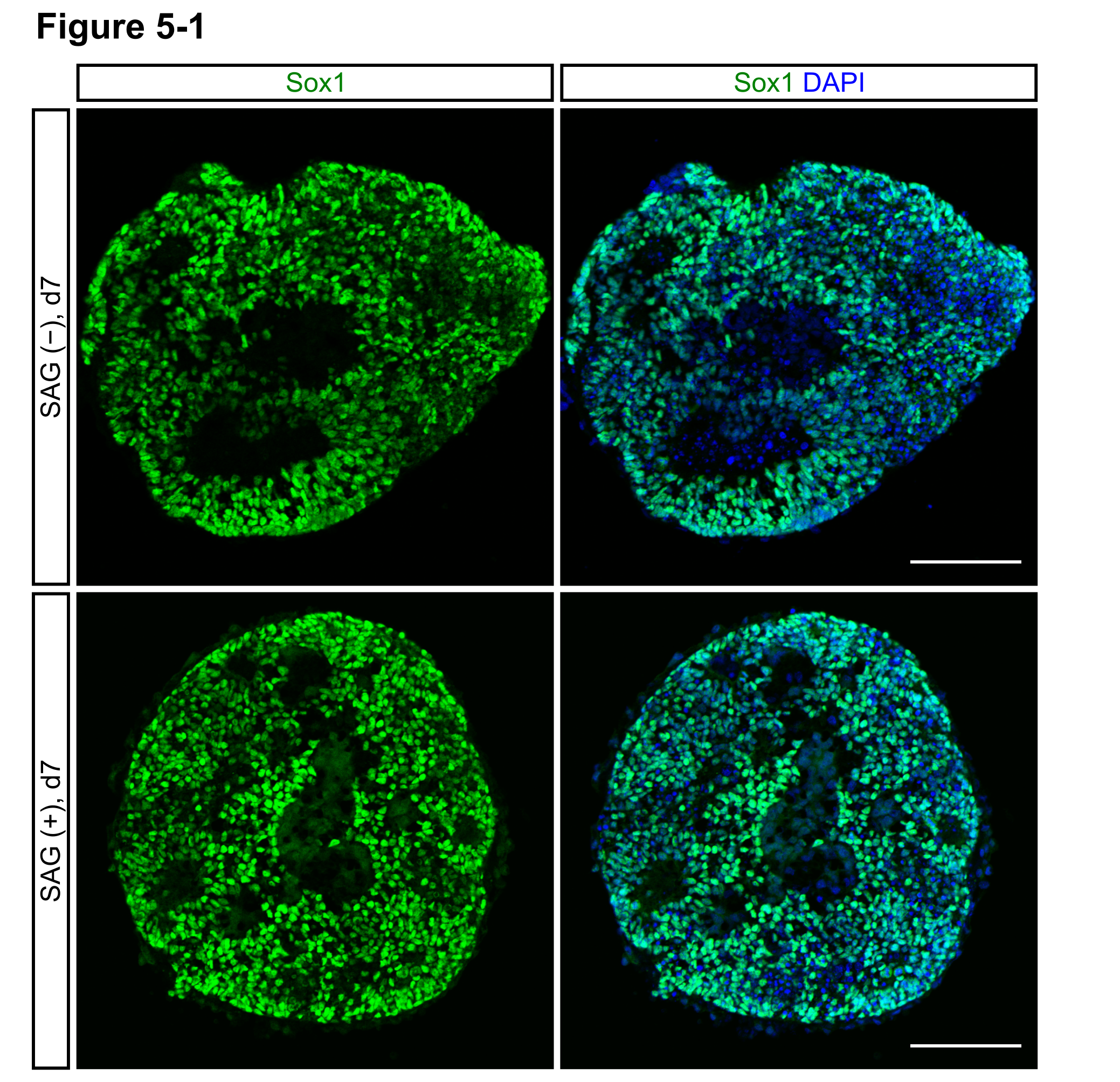

Supplement: Extended Data Figure 5-1 — Efficient induction of neural progenitors from mESCs in SFEBq culture with or without SAG. An SAG-treated (bottom) or untreated (top) mESC aggregate was immunostained for the general neural progenitor marker Sox1 on day 7. Nuclei were stained with DAPI. Scale bar: 100 μm. Download Figure 5-1, TIF file. [file enu-eN-NWR-0442-21-s02.tif]

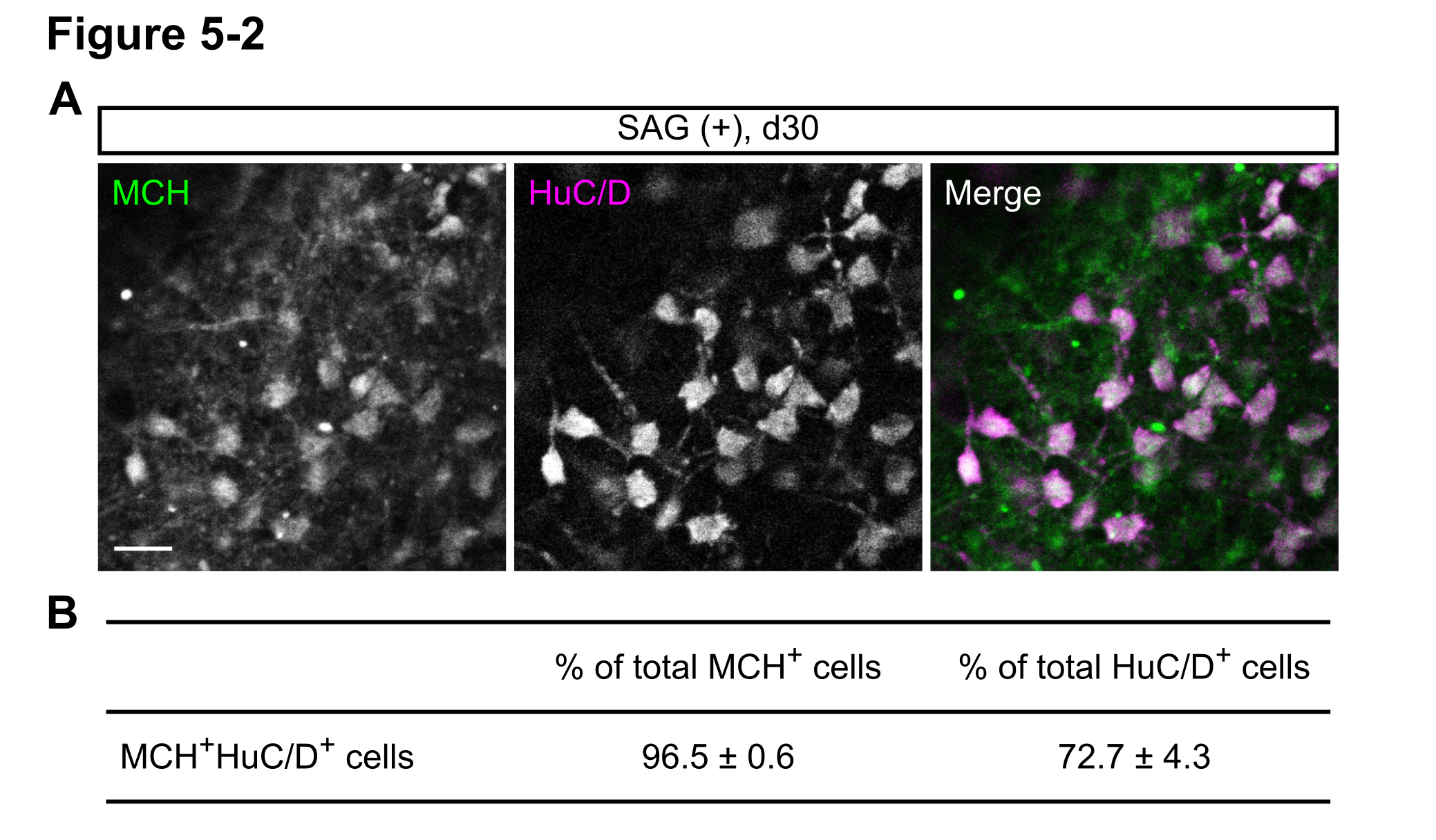

Supplement: Extended Data Figure 5-2 — Generation of MCH neurons in ES-Hypo in the presence of SAG. A, Representative images of an SAG-treated mESC aggregate immunostained for MCH and HuC/D on day 30. Scale bar: 20 μm. B, The percentage of MCH+HuC/D+ cells among total MCH+ or HuC/D+ cells. The values represent the mean ± SEM (n = 5 aggregates). Download Figure 5-2, TIF file. [file enu-eN-NWR-0442-21-s03.tif]

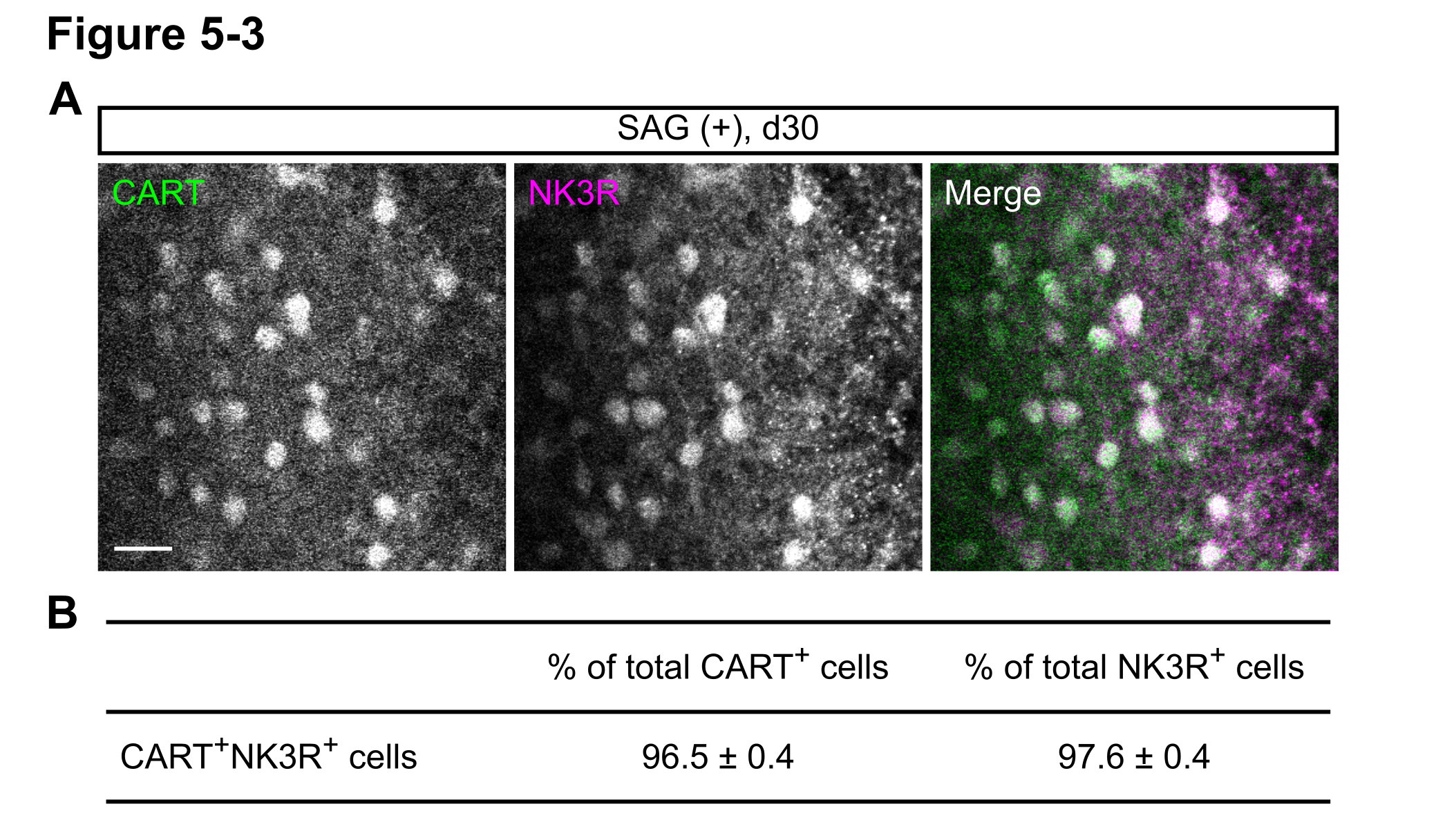

Supplement: Extended Data Figure 5-3 — Co-expression of CART and NK3R in ES-Hypo in the presence of SAG. A, Representative images of an SAG-treated mESC aggregate immunostained for CART and NK3R on day 30. Scale bar: 20 μm. B, The percentage of CART+NK3R+ cells among total CART+ or NK3R+ cells. The values represent the mean ± SEM (n = 4 aggregates). Download Figure 5-3, TIF file. [file enu-eN-NWR-0442-21-s04.tif]
